# ROS-induced PADI2 downregulation accelerates cellular senescence via the stimulation of SASP production and NFκB activation

**DOI:** 10.1007/s00018-022-04186-5

**Published:** 2022-02-26

**Authors:** Hyun-Jung Kim, Woo-Jin Kim, Hye-Rim Shin, Hee-In Yoon, Jae-I Moon, Eunji Lee, Jin-Muk Lim, Young-Dan Cho, Mi-Hye Lee, Hong-Gee Kim, Hyun-Mo Ryoo

**Affiliations:** 1 Department of Molecular Genetics & Dental Pharmacology, School of Dentistry and Dental Research Institute, Seoul National University, Seoul, South Korea; 2 Biomedical Knowledge Engineering Laboratory, School of Dentistry and Dental Research Institute, Seoul National University, Seoul, South Korea; 3 Alopax-Algo, Co. Ltd, Seoul, South Korea; 4 Department of Periodontology, School of Dentistry and Dental Research Institute, Seoul National University, Seoul, South Korea; 5 Department of Biology and Center for Cell Reprogramming, Georgetown University, Washington, DC USA

**Keywords:** Senescence, Reactive oxygen species, Peptidyl arginine deiminase 2, Senescence-associated secretory phenotype, NFκB, Osteoblast

## Abstract

Cellular senescence is closely related to tissue aging including bone. Bone homeostasis is maintained by the tight balance between bone-forming osteoblasts and bone-resorbing osteoclasts, but it undergoes deregulation with age, causing age-associated osteoporosis, a main cause of which is osteoblast dysfunction. Oxidative stress caused by the accumulation of reactive oxygen species (ROS) in bone tissues with aging can accelerate osteoblast senescence and dysfunction. However, the regulatory mechanism that controls the ROS-induced senescence of osteoblasts is poorly understood. Here, we identified Peptidyl arginine deiminase 2 (PADI2), a post-translational modifying enzyme, as a regulator of ROS-accelerated senescence of osteoblasts via RNA-sequencing and further functional validations. PADI2 downregulation by treatment with H_2_O_2_ or its siRNA promoted cellular senescence and suppressed osteoblast differentiation. CCL2, 5, and 7 known as the elements of the senescence-associated secretory phenotype (SASP) which is a secretome including proinflammatory cytokines and chemokines emitted by senescent cells and a representative feature of senescence, were upregulated by H_2_O_2_ treatment or *Padi2* knockdown. Furthermore, blocking these SASP factors with neutralizing antibodies or siRNAs alleviated the senescence and dysfunction of osteoblasts induced by H_2_O_2_ treatment or *Padi2* knockdown. The elevated production of these SASP factors was mediated by the activation of NFκB signaling pathway. The inhibition of NFκB using the pharmacological inhibitor or siRNA effectively relieved H_2_O_2_ treatment- or *Padi2* knockdown-induced senescence and osteoblast dysfunction. Together, our study for the first time uncover the role of PADI2 in ROS-accelerated cellular senescence of osteoblasts and provide new mechanistic and therapeutic insights into excessive ROS-promoted cellular senescence and aging-related bone diseases.

**Supplementary Information:**

The online version contains supplementary material available at 10.1007/s00018-022-04186-5.

## Introduction

Aging is a high risk factor for osteoporosis, which is characterized predominantly by declines in bone mass and strength. The consequent bone structure fragility is a primary cause of osteoporotic fracture in elderly people [1]. The histomorphometric analysis of bone biopsies obtained from the elderly showed that age-related bone loss is caused by impaired bone formation in comparison with bone resorption, indicating age-related osteoblast dysfunction as the main cause of age-related bone loss [2]. This osteoblast dysfunction can be resulted from extrinsic mechanism mediated by age-related changes in bone microenvironment including levels of hormones and growth factors, and/or intrinsic mechanisms caused by cellular senescence of osteoblasts [3]. Oxidative stress and damage by reactive oxygen species (ROS) formed exogenously or endogenously from normal metabolic processes increase during the aging process [4]. ROS are normally maintained at a balanced level by the cellular antioxidant systems. However, oxidative stress by the imbalance of ROS impairs skeletal integrity and reduces osteogenic differentiation of osteoblastic cells [5]. Furthermore, aberrant ROS can induce cellular senescence, and the accumulation of senescent cells with age influences the normal physiology of the tissues, causing progressive functional degeneration. Therefore, it is important to know the mechanism by which oxidative stress caused by the ROS accumulation leads to dysfunctions of osteoblasts during aging in order to refine therapeutic approaches to age-related bone loss.

Peptidylarginine deiminases (PADIs) are enzymes that convert positively charged peptidyl-arginine residues to neutral peptidyl-citrulline via a hydrolytic process termed citrullination or deimination [6]. This post-translational modification can induce profound changes in the structure and function of target proteins [6]. Five PADI enzymes (PADI1, 2, 3, 4 and 6) have been identified in mammals, and their expression pattern and function are tissue-specific [6, 7]. Citrullination has been shown to play important roles in multiple cellular processes such as inflammatory immune responses [8] and the regulation of gene expression [7]. The deregulation of PADIs is involved in the etiology of multiple human diseases, including autoimmune diseases and cancers [9]. However, the molecular function of PADIs in senescence and aging as well as in bones has not yet been reported.

Senescent cells secrete inflammatory cytokines, growth factors, chemokines, and proteases, termed the senescence-associated secretory phenotype (SASP) [10]. The composition of this special secretome is variable and depends on the triggers of senescence. The secretion of the SASP by senescent cells affects the tissue microenvironment in autocrine and paracrine manners, which causes the senescence to propagate to surrounding normal cells [11]. The key elements of the SASP are proinflammatory cytokines, including interleukin-1α (IL-1α), interleukin-6 (IL-6), and interleukin-8 (IL-8) [12]. CCL-2 (MCP-1), CCL-5 (RANTES), CCL-7 (MCP-3), CXCL-1, and CXCL12 (SDF-1) have also been identified as important components of the SASP of cells undergoing senescence [12]. As the master regulator of the SASP factors, activated NFκB p65/RelA binds the promoters of several SASP genes, thus regulating their expression during senescence or DNA damage response [13].

In this study, we aimed to explore the effects of oxidative stress on the senescence of osteoblasts and its regulatory mechanism to understand age-related bone loss. We elucidated for the first time the role of PADI2 as a key regulator for maintaining osteoblast function in oxidative stress-induced senescence of osteoblasts. We found that the reduction of PADI2 by ROS strongly induces cellular senescence and dysfunction of osteoblasts through the increase in SASP factors, especially, CCL2, 5, and 7. Blocking these SASP factors with neutralizing antibodies or siRNAs relieved ROS- or *Padi2* knockdown-induced senescence and dysfunction of osteoblasts. Furthermore, the increase of these SASP factors was mediated by the activation of NFκB and also the inhibition of NFκB by pharmacological inhibitor or siRNA significantly alleviated ROS- or *Padi2* knockdown-induced senescence and dysfunction of osteoblasts. Based on these findings, our study uncovered its novel regulatory mechanism by which oxidative stress induces osteoblast senescence and dysfunction, and also provide new insights for developing therapeutic treatments for age-associated bone loss.

## Materials and methods

### Reagents and antibodies

Here, 30% hydrogen peroxide solution (H1009), X-Gal (B4252), potassium hexacyanoferrate(III) (244023), and potassium ferrocyanide (P9387) were purchased from Sigma-Aldrich (St. Louis, MO, USA). Bay11-7082 (S2913) was purchased from Selleck Chemicals (Houston, TX, USA). Antibodies against the following proteins were used in this study: Padi2 (66386–1-Ig; Proteintech, Rosemont, IL, USA), α-tubulin (sc-8035; Santa Cruz Biotechnology, Inc., Dallas, Texas, USA), β-actin (sc-47778; Santa Cruz Biotechnology.), LaminA/C (sc-376248; Santa Cruz Biotechnology), p21 (#2947; Cell Signaling Technology, Inc., Danvers, MA, USA), mouse CCL2 antibody (AB-479-NA; R&D Systems, Inc, Minneapolis, MN, USA), mouse CCL5 antibody (AF478; R&D Systems), mouse CCL7 antibody (AF-456-NA; R&D Systems), and normal goat IgG control (AB-108-C; R&D Systems). The following siRNAs used in the present study were purchased from Origene (Rockville, MD, USA): Padi2 siRNA (SR418983B and C), RelA siRNA (SR427138A), Ccl2 siRNA (SR403037C), Ccl5 siRNA (SR400775A), Ccl7 siRNA (SR400957C), and negative control siRNA (SR30004).

### Cell culture

MC3T3-E1 cells were cultured in α-MEM with 10% fetal bovine serum (FBS) containing 100 U/mL penicillin and 100 µg/mL streptomycin. To induce osteoblast differentiation, α-MEM growth medium supplemented with 10 mM β-glycerophosphate and 50 µg/mL ascorbic acid was used. To mimic the chronic accumulation of ROS, 100 μM H_2_O_2_ was applied continuously during the entire cell culture period for osteoblast differentiation without withdrawal. The concentration of 100 μM H_2_O_2_ that does not show cytotoxicity while inhibiting osteoblast differentiation was determined through cell viability assay and ALP staining, respectively (data not shown). The medium was changed every 2–3 days with or without 100 μM H_2_O_2_ and/or 2.5 μM Bay11-7082. For RNA-seq, when cells were fully confluent, MC3T3-E1 cells were cultured for 1 and 4 days in the above-mentioned osteogenic medium with or without 100 μM H_2_O_2_. For siRNA experiments, cells were cultured to approximately 80 ~ 90% confluence, and then transfected with 20 nM siRNA each or combination using lipofectamine RNAiMax reagent (#13778; Invitrogen, Carlsbad, CA, USA) according to the manufacturer’s instructions. When transfected cells reached full confluence, cells were treated with or without 100 μM H_2_O_2_ or 2.5 μM Bay11-7082 during indicated time periods for each experiment. Treatment time of H_2_O_2_ and culture periods for each experiment were indicated in figure legends. For long-term culture for osteoblast differentiation, osteogenic medium was changed every 2–3 days with or without 100 μM H_2_O_2_ and/or 2.5 μM Bay11-7082. Human mesenchymal stem cells were purchased from STEMCELL Technologies (Vancouver, Canada) and cultured in accordance with the manufacturer’s protocol.

### Alkaline phosphatase staining

The detailed procedure of each cell culture was explained above and figure legends. Alkaline phosphatase (ALP) staining was performed with alkaline phosphatase staining kit (Cat# AK20; COSMO BIO Co. Ltd, Japan) according to the manufacturer’s instructions. In brief, cells were carefully rinsed with 1xPBS and fixed with 4% paraformaldehyde at room temperature for 15 min. After fixation, cells were rinsed with PBS and then incubated in BCIP/NBT liquid substrate for 30 min–2 h. Color change was monitored and reaction was stopped with washing with PBS. Stained cell cultures were imaged by FUSION FX (VILBER, France). For quantification of ALP staining, the intensity of staining in the region of interest (ROI) was analyzed and expressed as % area using ImageJ software (NIH). All the staining data were confirmed by three repeated experiments.

### Alizarin red S staining

The detailed procedure of cell culture was explained above and figure legends. After induction of osteoblast mineralization, cells were fixed with 4% paraformaldehyde at room temperature for 15 min. Cells were rinsed with 1xPBS and stained with 500 μL 0.5% alizarin red stain solution, pH 4.2, for 30 min at room temperature. After incubation, cells were rinsed with ddH_2_O on an orbital shaker for 5 min, three times. The mineralized nodules were stained as red spots after removal of unincorporated excess dye with ddH_2_O. Plates were scanned with FUSION FX (VILBER, France). For quantification of ARS staining, stained nodules in the ROI was analyzed and expressed as a percentage of the mineralized area fraction to the total ROI using ImageJ software. All the staining data were confirmed by three independent experiments.

### SA-β-Gal staining and quantification

SA-β-Gal staining was performed as previously described [14]. Cells were fixed for 10 min at room temperature in 4% paraformaldehyde. After washing the cells three times with PBS, they were stained at 37 °C overnight in a non-CO_2_ incubator in staining solution [40 mM Na_2_HPO_4_, pH6.0, 150 mM NaCl, 2 mM MgCl_2_, 5 mM K_3_Fe(CN)_6_, 5 mM K_4_Fe(CN)_6_, and 1 mg/ml X-Gal]. After washing three times with PBS, cells were permeabilized for 15 min at room temperature with 0.2% Triton X-100 in PBS. Mounting medium containing 4′,6-diamidino-2-phenylindole (DAPI) was used to visualize the nucleus (AR-6501-01; ImmunoBioScience Corp., Mukilteo, WA, USA). Images were acquired using an Eclipse TS-100 inverted microscope (Nikon). DAPI-stained nucleus and SA-β-Gal-positive cells were quantified by ImageJ software. Percentage of SA- β -Gal-positive cells in each group was calculated as the percentage of SA-β-Gal-positive cells relative to the total cells stained with DAPI in the same field. For the statistics, images were acquired at 20 × magnification and each group was measured with 8 ~ 10 fields. To avoid bias in the selection of SA-β-Gal-positive cells, the region of DAPI-stained cells was first imaged, followed by the obtaining of a SA-β-Gal-stained image of the same region. Three independent experiments were performed with triplicate.

### Western blot analysis

Protein lysates were prepared using a PRO-PREP protein extraction solution (Cat#17081, iNtRON, South Korea), in accordance with the manufacturer’s protocol. Equal amounts of protein were resolved by sodium dodecyl sulfate polyacrylamide gel electrophoresis (SDS-PAGE) and transferred onto a polyvinylidene fluoride (PVDF) membrane. After the process of blocking with 5% nonfat skim milk, the membrane was blotted with designated primary and secondary antibodies, developed using the enhanced chemiluminescence method (Clarity™ Western ECL Substrate, #170–5060; Bio-Rad), and visualized with FUSION FX (VILBER, France). α-Tubulin or β-actin was used as a protein loading control.

### RNA preparation and quantitative real-time PCR

Total RNA was isolated using the RNeasy Mini Kit (Qiagen, Hilden, Germany). Total RNA (1 μg) was reverse-transcribed into cDNA using the PrimeScript RT Master Mix (Perfect Real Time) (RR036A; TaKaRa, Japan) and real-time quantitative PCR (qPCR) was performed using the TB Green® Premix Ex Taq™ (RR420A; TaKaRa) on a StepOnePlus™ Real-Time PCR System (Applied Biosystems™). The following thermal conditions were used for real-time PCR: 95 °C for 1 min, followed by 40 cycles of 95 °C for 15 s and 60 °C for 30 s. The primers used in this study are listed in Supplementary Table S5. The relative expression levels were calculated with the 2^−*ΔΔCT*^ method [15]. The reaction was carried out using three sample replicates with three independent experiments.

### ELISA

The levels of CCL2, CCL5, and CCL7 released into the conditioned medium were measured using mouse CCL2 ELISA kit (ab208979; Abcam, Cambridge, UK), mouse CCL5 ELISA kit (ab100739; Abcam), and mouse CCL7 ELISA kit (ab205571; Abcam), in accordance with the manufacturer’s protocols.

### Nuclear-cytoplasmic fractionation

Cells were collected in phosphate-buffered saline (PBS), and the cell pellet was divided equally between two tubes for whole-cell lysate and cell fractionation. Whole-cell lysates were prepared with 100 μl of lysis buffer containing 25 mM Tris–HCl pH 7.4, 150 mM NaCl, 1% NP-40, 1 mM EDTA, 5% glycerol, protease inhibitor cocktail (Roche, #11–836-153-001), and phosphatase inhibitor cocktail 2 and 3 (Sigma-Aldrich, P5726 and P0044). Nuclear-cytoplasmic fractionation was conducted using NE-PER™ nuclear and cytoplasmic extraction reagents (78855; Life Technologies, Carlsbad, CA, USA), in accordance with the manufacturer’s instructions. The protein concentration was determined using a Pierce™ BCA Protein Assay Kit (Thermo Fisher Scientific). Whole-cell lysates, cytoplasmic extracts, and nuclear extracts were resolved by SDS-PAGE, transferred to a PVDF membrane, and the membranes were probed with the designated primary and secondary antibodies. Tubulin and Lamin A/C were used as cytoplasmic and nuclear markers, respectively.

### Immunofluorescence staining and confocal microscopy

Cells grown on coverslips were fixed in 4% paraformaldehyde, blocked, and incubated with primary and corresponding secondary antibodies (Alexa Fluor 488- or 568-conjugated) (A11034; Invitrogen). Mounting medium containing DAPI was used to visualize the nucleus. The cells were examined using a confocal microscope (LSM 800, Carl Zeiss) and representative cells were selected and imaged.

### Quantification of γH2AX foci

After immunofluorescence staining, at least 30 images per each group were randomly acquired with 40 × objective for foci counting. The number and intensity of γH2AX foci in DAPI-stained nucleus was analyzed using ImageJ software. Cells with ≥ 10 foci with an intensity (≥ 10,000) are considered as γH2AX foci-positive cells. Percentage of γH2AX foci-positive cells was calculated as the percentage of γH2AX-positive cells out of the total cells analyzed per group. More than 100 nuclei per group were analyzed in each independent experiment. Two independent experiments were performed.

### mRNA-seq and data analysis

RNA quality was assessed by analyzing rRNA band integrity on an Agilent RNA 6000 Nano kit (Agilent Technologies, Santa Clara, CA, USA). Ahead of cDNA library construction, magnetic beads with oligo(dT) were used on 1 μg of total RNA to enrich poly(A) mRNA from it. Then, the purified mRNAs were disrupted into short fragments and the corresponding double-stranded cDNAs were immediately synthesized. These cDNAs were then subjected to end-repair and poly(A) addition, and connected with sequencing adapters using the TruSeq Stranded RNA Sample Prep Kit (Illumina). The suitable fragments automatically purified using a BluePippin 2% agarose gel cassette (Sage Science, MA) were selected as templates for PCR amplification. The final library sizes and qualities were evaluated electrophoretically with an Agilent High Sensitivity DNA kit (Agilent Technologies) and the fragments were found to be between 350 and 450 bp. Subsequently, the library was sequenced using an Illumina NovaSeq6000 sequencer (Illumina) and paired-end (2 × 100 bp) sequencing was performed at TheragenEtex Bio Institute (Seoul, South Korea). Raw data quality control was performed using FastQC, and sequencing data were first aligned to the *Mus musculus* GRCm38 reference genome using HISAT 2.1.0 aligner [16]. The expression read count value was extracted by applying StringTie 2.0 [17]. Differentially expressed genes were selected with the following criteria: fold change of 1.5, log2-normalized read count of ≥ 5 to minimize false counts, and *P* < 0.05 with *t*-test statistics. Gene Ontology analysis was performed using the DAVID v6.8 functional annotation online tool.

### Statistical analysis

To ensure data reliability, all experiments were performed as at least two or three independent experiments with three replicates and representative results are shown in figures. For statistical analyses, *P* values were calculated by unpaired two-tailed Student’s *t-*test (when comparing only two groups) or one-way ANOVA or two-way ANOVA (when comparing more than two groups) in GraphPad Prism 9. All results are expressed as the mean ± SD, and differences were considered significant at *P* < 0.05. *P* values are as follows: *P < 0.05; **P < 0.01; ***P < 0.001; ****P < 0.0001.

## Results

### ROS induce the senescence of osteoblast cells and downregulate PADI2 levels

**Fig. 1:**
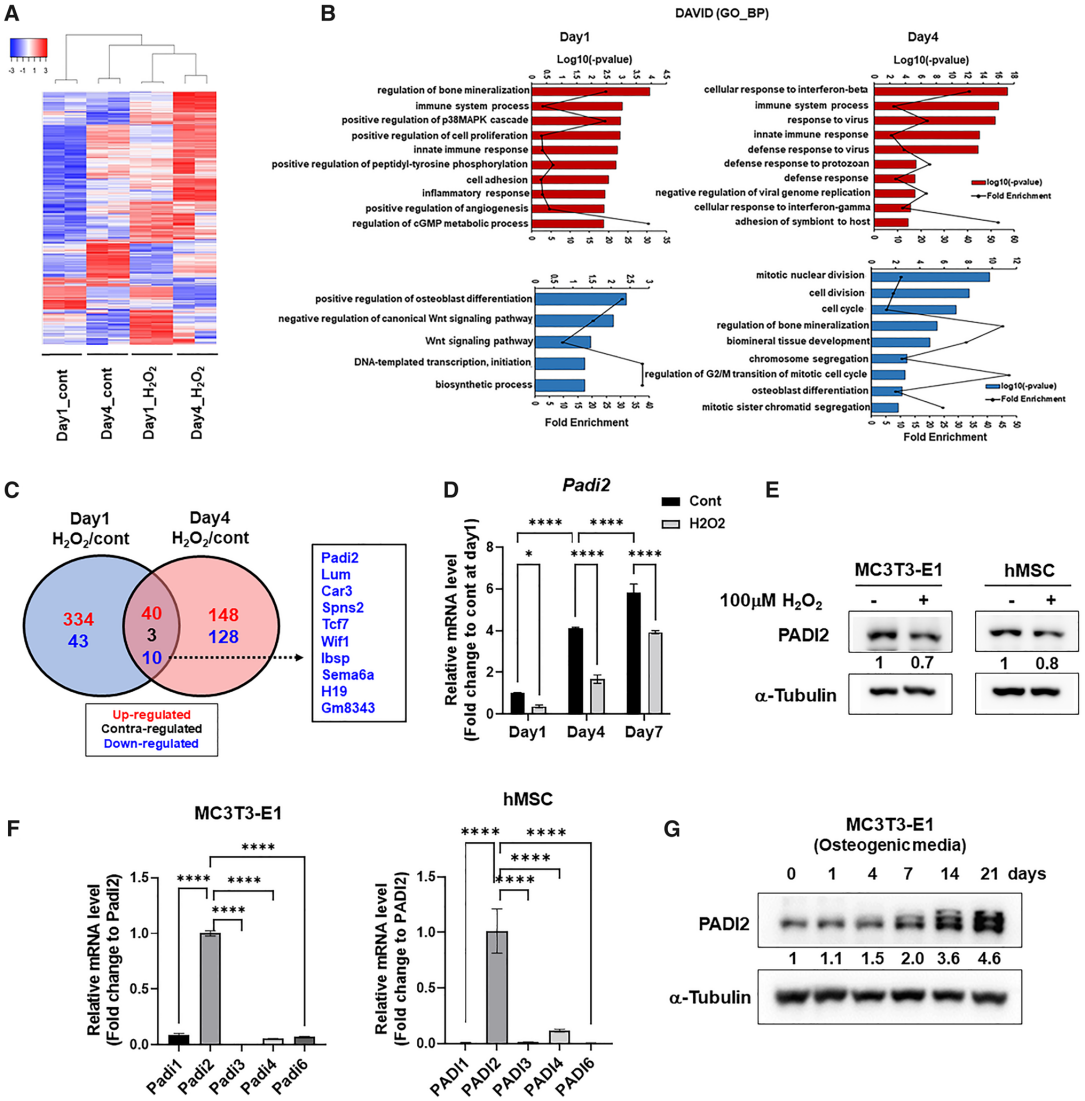
ROS induce the reduction of PADI2 level in murine- and human-derived mesenchymal lineage cells. **A** Hierarchical clustering analysis of 706 DEGs from MC3T3-E1 cells treated with or without 100 μM H_2_O_2_ for 1 or 4 days in osteogenic medium (fold change, 1.5; log2-normalized read count of ≥ 5; *P* < 0.05). **B** DAVID GO term analysis for biological processes of the upregulated (Red) or downregulated (Blue) genes on each day. **C** The Venn diagram shows the numbers of DEGs with shared and unique expression between day 1 and day 4. Commonly downregulated genes are listed in the right box. **D** MC3T3-E1 cells were treated with or without 100 μM H_2_O_2_ in the osteogenic medium for each indicated period. The Padi2 mRNA level was determined by RT-qPCR and the fold change in mRNA levels was calculated by normalization to *Gapdh*. Data are presented as the mean of three replicates; bars, ± SD; statistical significance was determined using two-way ANOVA for multiple comparisons, *P < 0.05, ****P < 0.0001. **E** Western blot analysis of PADI2 in MC3T3-E1 and hMSC treated with or without 100 μM H_2_O_2_ for 24 h. Protein level is quantified using ImageJ and normalized with a-Tubulin. After normalization, fold changes of treated /non-treated control are presented. **F** RT-qPCR of *PADI* isoforms in MC3T3-E1 and hMSC. Data are presented as the mean of three replicates; bars, ± SD; statistical significance was determined using one-way ANOVA for multiple comparisons, ****P < 0.0001. **G** Western blot analysis of PADI2 expression levels at the indicated time points during osteogenic differentiation of MC3T3-E1. Protein level is quantified using ImageJ and normalized with a-Tubulin. After normalization, fold changes to day0 are presented

In agreement with the clinical and epidemiological studies that aging per se is a pivotal determinant of the decline of bone mass and strength [18, 19], several lines of evidence strongly suggest that the oxidative stress which underlies physiological organismal aging is an important pathogenic mechanism of age-related bone loss [20, 21]. Excessive ROS impair skeletal integrity and reduce osteogenic differentiation of osteoblastic cells [5], and also that chronic imbalance of ROS can induce cellular senescence contributing to tissue aging. We first confirmed whether oxidative stress induces the senescence and dysfunction of osteoblasts in our experimental setting. SA-β-Gal staining, the most representative senescence marker, showed that the treatment of 50 μM or 100 μM H_2_O_2_ for 24 h induced the senescence of MC3T3-E1 osteoblasts in a dose-dependent manner (Supplementary Fig. S1A). Because these concentrations of H_2_O_2_ did not cause cytotoxicity even after long-term treatment, the H_2_O_2_ treatment concentration was determined to be 100 μM in all subsequent experiments. The *Cdkn1a* mRNA and the protein level of its translation product p21, a representative molecular marker for senescence, were also significantly increased by H_2_O_2_ treatment for 1 and 4 days (Supplementary Fig. S1B and C). The expression of *Cdkn2A* (p16^INK4a^) and *Cdkn2B* (p15^INK4b^), which are other representative senescence molecular markers, was not basally expressed, nor was it induced by H_2_O_2_ treatment in MC 3T3-E1 cells (data not shown). In consistence with the induction of senescence by H_2_O_2_ treatment, alkaline phosphatase (ALP) and alizarin Red S (ARS) staining showed that the chronic treatment of H_2_O_2_ significantly inhibited the osteogenic differentiation of MC3T3-E1 cells (Supplementary Fig. S1D). These results indicate that chronic exposure of excessive ROS cause the senescence and dysfunction of osteoblasts. Next, to identify key regulators directly involved in ROS-induced senescence and dysfunction of osteoblasts, an RNA-seq analysis was performed with MC3T3-E1 cells treated with H_2_O_2_ for 1 and 4 days in osteogenic medium and matching untreated control cells. In total, 706 differentially expressed genes (DEGs) satisfying the criteria of fold change 1.5, normalized log2 read count ≥ 5, and *P* < 0.05 in day 1 or day 4 groups were selected and listed (Supplementary file 2). The heat map with 706 DEGs shows that the trend in gene expression change was consistent in the samples within each group (*n* = 2 per group) and the differences between untreated and H_2_O_2_-treated groups were statistically significant and hierarchically well clustered (Fig. 1A). To systematically characterize the biological processes associated in H_2_O_2_-induced senescence of osteoblasts according to the H_2_O_2_ treatment periods (Days 1 and 4), we performed Gene Ontology (GO) analysis of DEGs on days 1 and 4 using the DAVID functional annotation online tool (Fig. 1B and Supplementary files 3, 4, 5 and 6). Interestingly enough, genes that showed increased expression in response to H_2_O_2_ on both days were significantly associated with terms related to immune responses, including the immune system process, the innate immune response, and the inflammatory response. Meanwhile, genes with downregulated expression on Day 1 were particularly associated with biological processes related to osteoblast differentiation and the Wnt signaling pathway. Genes with a decrease in expression on Day 4 were mainly associated with biological processes, including cell division and cell cycle, as well as bone mineralization, which were significantly correlated with senescence (Fig. 1B). In further support, Gene Set Enrichment Analysis (GSEA) showed that one gene set (Cell Signaling by Wnt) among upregulated gene sets on Day1 cont group was significant at FDR < 25%, suggesting that gene expression profile of H_2_O_2_-treated group may be negatively associated with this gene set (Supplementary Fig. S2). In comparison gene expression profile of Day4 H_2_O_2_ with its control, 44 gene sets (FDR < 25%) including skeletal system development, chromosome segregation, cell cycle, cell division, and mitotic nuclear division were negatively correlated with H_2_O_2_ treatment (Supplementary Fig. S3 and Supplementary files 7). 26 gene sets including skeletal system development, chromosome segregation, cell cycle, cell division, and mitotic nuclear division had significant positive correlation with H_2_O_2_ treatment at FDR < 25% (Supplementary Figure S3 and Supplementary files 8). We compared the gene set regulated by H_2_O_2_ treatment for 1 day with that regulated by 4 day H_2_O_2_ treatment. Among a total of 706 genes, 40 genes were commonly upregulated and 10 genes were commonly downregulated by the H_2_O_2_ treatment on both days (Fig. 1C, Supplementary Tables S1 and S2). Three genes showed regulation in the opposite directions on these two days (Fig. 1C, Supplementary Table S3). We performed RT-qPCR for validating 8 protein coding genes (*Padi2*, *Lum*, *Car3*, *Spns2*, *Tcf7*, *Wif1*, *Ibsp*, and *Sema6a*) among commonly downregulated 10 genes (Supplementary Fig. S4). Consistent with RNA-seq results, five genes of *Padi2*, *Car3*, *Tcf7*, *Sema6a* and *Spns2* were significantly reduced by both 1 and 4 day H_2_O_2_ treatments. The remaining three genes (*Lum, Ibsp,* and *Wif1*) were significantly decreased in the 4 day-treatment group, but tended to be down-regulated without significance in the 1 day treatment group. PADI2 is a unique post-translational modification enzyme capable of converting positive charged arginine to neutral charged citrulline and affecting protein structure, function and protein–protein interaction [6]. It was reported that the expression and citrullination of PADI2 decrease with age in the murine retina [22]. *Padi2* is also significantly downregulated at both H_2_O_2_ treatment groups in our RNA-seq and RT-qPCR data. For these reasons, we selected *Padi2* as a candidate regulator in ROS-induced senescence of osteoblasts and further validated its function in ROS-accelerated senescence and dysfunction of osteoblasts. We confirmed that H_2_O_2_ treatment for 1, 4, and 7 days on MC3T3-E1 cells significantly downregulated both *Padi2* mRNA (Fig. 1D). Western blot analysis also showed that PADI2 protein level also downregulated by H_2_O_2_ treatment (Fig. 1E). Moreover, the downregulation of PADI2 by H_2_O_2_ treatment was also observed in human mesenchymal stromal cells (hMSCs) (Fig. 1E). Next, we checked the expression levels of the five PADI isoforms in both MC3T3-E1 cells and hMSCs to determine which isoforms are prevalent in these cells. Interestingly, RT-qPCR revealed that *Padi2* was the predominant isoform in both MC3T3-E1 and hMSCs (Fig. 1F). These results suggest that the H_2_O_2_-induced reduction of PADI2 expression is a common phenomenon in both mouse- and human-derived mesenchymal lineage cells, and PADI2 is a main PADI isoform that plays a key role in osteoblasts as well as MSCs. Consistent with the gradual increase in basal Padi2 mRNA levels during osteoblast differentiation (Fig. 1D), western blot analysis also showed that PADI2 protein levels progressively increased during osteoblast differentiation (Fig. 1G). Interestingly, PADI2 band was singular at the beginning of differentiation, then exhibited up to 3 bands as differentiation processed (Fig. 1G). These results imply that PADI2 plays an important role at the stage of osteoblast differentiation and maturation and also post-translational modifications of PADI2 may have a role in PADI2 function and osteoblast differentiation.

### The reduction of PADI2 causes the senescence of osteoblasts and hinders osteogenic differentiation

**Fig. 2:**
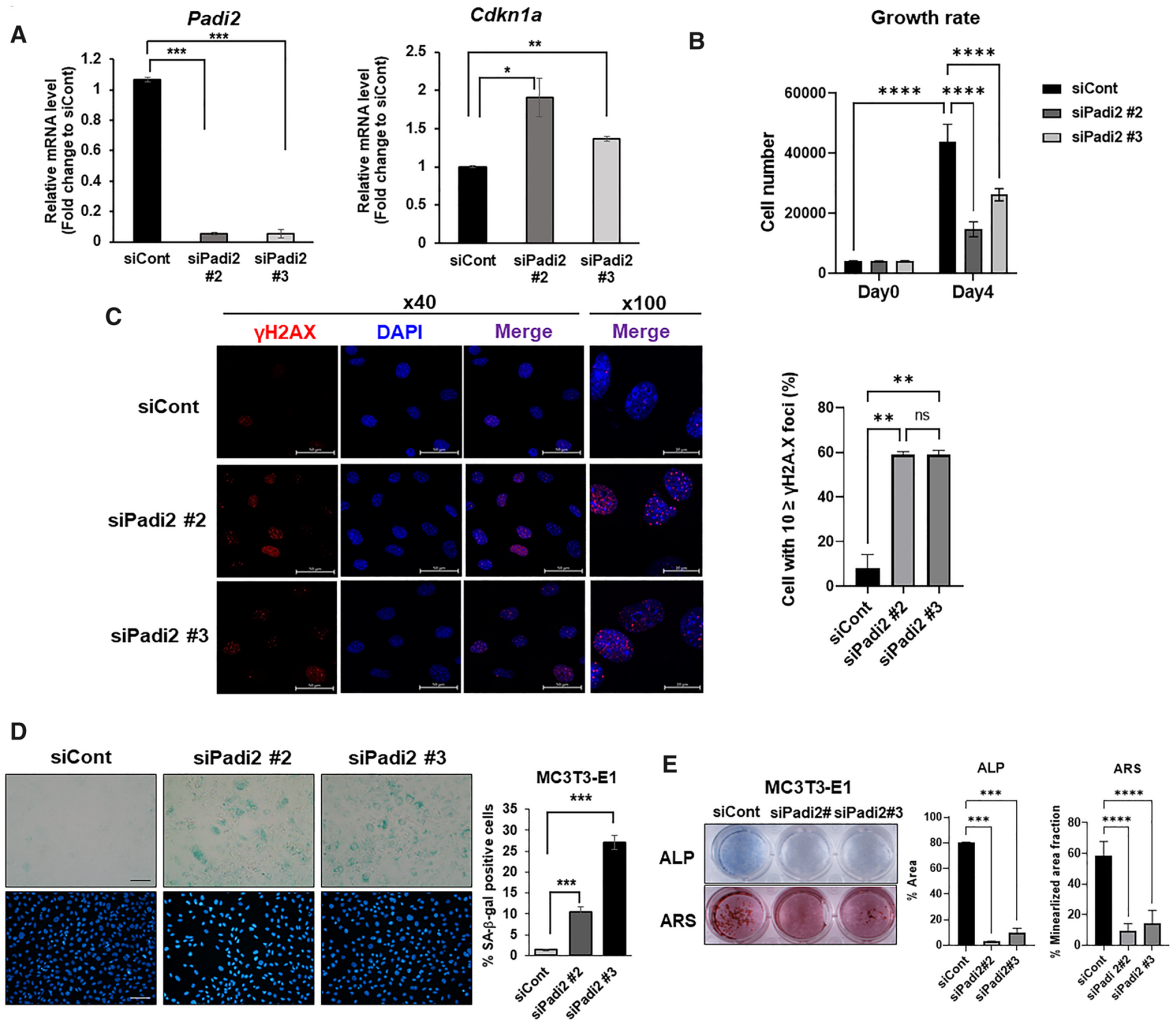
*Padi2* knockdown accelerates cellular senescence and functional decline of osteoblasts. **A** MC3T3-E1 cells were transfected with scrambled control siRNA (siCont), Padi2 siRNA #2 (siPadi2 #2), or Padi2 siRNA #3 (siPadi2 #3) and then incubated for 72 h. The mRNA levels of *Padi2* and *Cdkn1a* were determined by RT-qPCR and the fold change in mRNA levels was calculated by normalization to *Gapdh*. Data are presented as the mean of three replicates; bars, ± SD; statistical significance was determined using one-way ANOVA for multiple comparisons, *P < 0.05, **P < 0.01, ***P < 0.001. Three independent experiments were performed. **B** A total of 4×10^3^ cells transfected with each siRNA were seeded on 24-well plates on day 0 and then cells were cultured in growth medium and counted on day 4. Data are presented as the mean of three replicates; bars, ± SD; statistical significance was determined using two-way ANOVA for multiple comparisons, ****P < 0.0001. Three independent experiments were performed. **C** Representative confocal microcopy images of γH2AX immunostaining. Cells were plated on coverslips to approximately 40–50% confluence 48 h after MC3T3-E1 cells were transfected with each siRNA and then incubated for additional 24 h, followed by γH2AX staining (Red). The nuclei were stained with DAPI (Blue). Scale bar, 50 μm for magnification × 40, 20 μm for magnification × 100. In the graph on the right, the number of cells with ≥ 10 γH2AX foci was presented as a percentage. Bars, mean ± SD of two independent experiments; statistical significance was determined using two-way ANOVA for multiple comparisons, **P < 0.01; ns, not significant. **D** Representative images of SA-β-Gal staining (upper panel) of MC3T3-E1 transfected with siCont or siPadi2. Transfected cells were cultured for 4 days and SA-β-Gal staining was performed after fixation with 4% paraformaldehyde. Nuclei were stained with DAPI (lower panel). SA-β-Gal-positive cells in each group were calculated as the percentage of SA-β-Gal-positive cells relative to the total cells stained with DAPI in the same field (10 fields/group). Magnification ×20, Scale bars 100 μm. Bars, ± SD; statistical significance was determined using one-way ANOVA for multiple comparisons, ***P < 0.001. Three independent experiments were performed. **E** MC3T3-E1 cells transfected with siCont or siPadi2 were cultured in osteogenic medium for 4 and 18 days for ALP and ARS staining, respectively. Quantification of each staining was performed by ImageJ. Bars, mean ± SD; statistical significance was determined using one-way ANOVA for multiple comparisons, ***P < 0.001, ****P < 0.0001. Three independent experiments were performed

To investigate whether PADI2 is a critical component of the ROS-induced cellular senescence and dysfunction of osteoblasts, the *Padi2* gene was knocked down with small interfering RNAs (siRNAs) specifically targeting *Padi2* in MC3T3-E1 cells (Fig. 2A). *Padi2* knockdown significantly induced *Cdkn1a* gene expression in MC3T3-E1 cells (Fig. 2A) and the growth rate showed a significant decrease in *Padi2*-knocked-down cells compared with that in siCont-transfected MC3T3-E1 cells (Fig. 2B). Senescing cells typically accumulate γH2AX foci associated with DNA damage response (DDR) elicited by intrinsic and extrinsic stress such as ROS and ionizing radiation [23, 24]. The accumulation of γH2AX foci in cell cultures has been correlated with in vivo observations in somatic and germ tissues of aging mice [24]. Therefore, we investigated whether *Padi2* knockdown induces the accumulation of γH2AX foci. Both types of *Padi2* siRNA knockdown significantly increased γH2AX foci by approximately 60% (Fig. 2C). We also established *Padi2* knockout (KO) cell lines of MC3T3-E1 (#3–4 and #5–6) using the CRISPR–Cas9 gene editing system and confirmed that PADI2 was completely absent from both #3–4 and #5–6 *Padi2* KO cell lines, in contrast to the wild type, by western blot analysis (Supplementary Figure S5A). Consistent with the findings upon *Padi2* knockdown (Fig. 2B), BrdU assay showed that the proliferation rate significantly decreased in both *Padi2* KO cell lines compared with that in the wild type (Supplementary Fig. S5B). Cells with the accumulation of γH2AX foci also dramatically increased in *Padi2* KO cells by about 80% compared with wild type cells (~ 10%) (Supplementary Figure S5C). To determine whether the loss of PADI2 induces cellular senescence of osteoblasts, SA-β-Gal staining was performed in *Padi2*-knockdown MC3T3-E1 cells. SA-β-Gal-positive cell number significantly increased in *Padi2*-knockdown cells compared with that in siCont-transfected cells (Fig. 2D). As was consistent with this finding, the *Padi2* KO cell lines also showed a much higher increase in the number of SA-β-Gal-positive cells (Supplementary Fig. S5D). ALP and ARS staining showed that the loss of *Padi2* drastically inhibited the osteogenic differentiation of MC3T3-E1 cells (Fig. 2E and Supplementary Fig. S5E). Pharmacological inhibition of PADI2 using Cl-amidine, a pan-PADI inhibitor, also suppressed the osteogenic differentiation of MC3T3-E1 cells (Supplementary Fig. S5F), suggesting the importance of PADI2-mediated protein citrullination for cellular senescence and osteogenic differentiation. Taken together, these results strongly indicate that a decrease in PADI2 induces osteoblast senescence accompanied by a dysfunction of osteoblasts.

### Excessive ROS or *Padi2* knockdown promotes CCL2, 5, and 7 proinflammatory SASP production

**Fig. 3:**
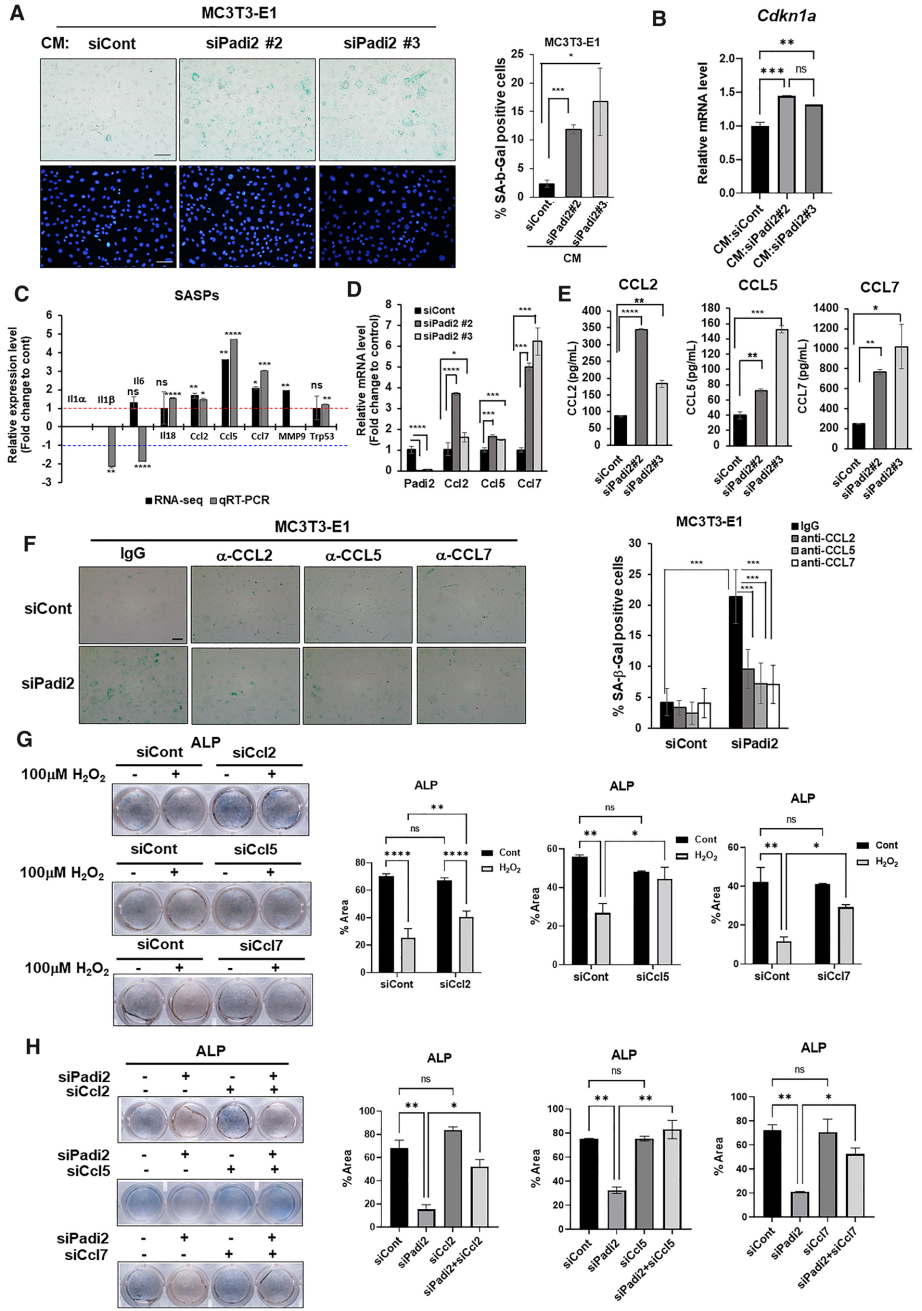
Upregulation of the SASP by ROS and *Padi2* knockdown leads to cellular senescence and the inhibition of the SASP ameliorates cellular senescence and osteogenic dysfunction. **A** Representative images of SA-β-Gal staining. MC3T3-E1 cells were cultured in the conditioned medium (CM) from MC3T3-E1 transfected with siCont or siPadi2 for 8 days and SA-β-Gal staining was performed. Percentage of SA-β-Gal-positive cells in each group was calculated as the percentage of SA-β-Gal-positive cells relative to the total cells stained with DAPI in the same field (8–10 fields/group). Magnification ×20, Scale bars 100 μm. Bars, statistical significance was determined using one-way ANOVA for multiple comparisons, *P < 0.05, ***P < 0.001. Three independent experiments were performed. **B** MC3T3-E1 cells were cultivated under Padi2 KD-CM for 8 days or siCont-CM, followed by performing RT-qPCR of *Cdkn1a.* Data for RT-qPCR are presented as the mean of three replicates; bars, ± SD. Statistical significance was determined using one-way ANOVA for multiple comparisons, **P < 0.01, ***P < 0.001; n.s., not significant. Two independent experiments were performed. **C** Analysis of the relative expression levels of the SASP genes by RNA-seq data (black bar) and RT-qPCR (gray bar). Data are the fold change of 4 day-H_2_O_2_-treated group relative to the non-treated control. The red and blue-dotted lines mean fold change 1 and − 1, respectively. Data for RT-qPCR are presented as the mean of three replicates; bars, ± SD. statistical significance was determined using two-tailed Student’s t-test, *P < 0.05, **P < 0.01, ***P < 0.001, ****P < 0.0001; n.s., not significant. Three independent experiments were performed for RT-qPCR. **D** MC3T3-E1 cells were transfected with siCont, siPadi2 #2 or siPadi2 #3 and then incubated for additional 4 days in the osteogenic medium. The expression level of each gene was measured by RT-qPCR and the fold change in mRNA levels was calculated by normalization to *Gapdh*. Data are presented as the mean of three replicates; bars, ± SD; statistical significance was determined using one-way ANOVA for multiple comparisons, *P < 0.05, ***P < 0.001, ****P < 0.0001. Three independent experiments were performed. **E** Five days-cell culture soup from MC3T3-E1 cells transfected with siCont, siPadi2 #2, or siPadi2 #3 was collected and the levels of CCL2, CCL5, and CCL7 in each cell culture soup were measured by ELISA. Data are presented as the mean of three replicates; bars, ± SD; statistical significance was determined using one-way ANOVA for multiple comparisons, *P < 0.05, **P < 0.01, ***P < 0.001, ****P < 0.0001. Three independent experiments were performed. **F** Representative images of SA-β-Gal staining. MC3T3-E1 cells knocked down with siCont or siPadi2 were treated with IgG or each neutralizing antibody for 4 days and SA-β-Gal staining was performed. Quantity of SA-β-Gal-positive cells was expressed as the percentage of SA-β-Gal-positive cells relative to the total cells stained with DAPI in the same field (8–10 fields/group). Magnification ×20, Scale bars 100 μm. bars, mean ± SD. statistical significance was determined using two-way ANOVA for multiple comparisons, ***P < 0.001. Three independent experiments were performed. **G** When MC3T3-E1 cells transfected with siCont, siCcl2, siCcl5, or siCcl7 were fully confluent, cells were treated with 100 μM H_2_O_2_ for 4 days in osteogenic medium and ALP staining was performed. Quantification of ALP staining was performed by ImageJ. Bars, mean ± SD; statistical significance was determined using one-way or two-way ANOVA for multiple comparisons, *P < 0.05, **P < 0.01, ****P < 0.0001; ns, not significant. **H** When MC3T3-E1 cells knocked down with siPadi2 in combination with or without siCcl2, 5, or 7 were fully confluent, cells were changed with osteogenic medium and incubated for 4 days and ALP staining was performed. Quantification of ALP staining (right). Bars, mean ± SD; statistical significance was determined using one-way or two-way ANOVA for multiple comparisons, *P < 0.05, **P < 0.01,; *ns* not significant. Three independent experiments were performed

Inflammatory and immune-modulatory cytokines and chemokines secreted by senescent cells can extend cellular senescence and alter the microenvironment to the direction favorable for senescence [25]. Our data also showed that the gene set with increased expression in response to H_2_O_2_ showed the significant enrichment of terms related to immune responses including inflammatory responses in our RNA-seq data, suggesting that proinflammatory SASP factors might be involved in H_2_O_2_ treatment or *Padi2* knockdown-induced senescence of osteoblasts (Fig. 1B, Supplementary Fig. S3 and Supplementary files 8). To confirm the involvement of the SASP, we investigated whether the conditioned medium derived from the *Padi2*-knockdown MC3T3-E1 cells (Padi2 KD-CM) can induce the senescence of MC3T3-E1 cells. Interestingly, the culture of MC3T3-E1 cells under Padi2 KD-CM for 8 days significantly promoted the senescence of MC3T3-E1 cells compared with siCont-CM (Fig. 3A). Consistently, the *Cdkn1a* mRNA level significantly increased in MC3T3-E1 cells cultivated under Padi2 KD-CM for 8 days compared with those under siCont-CM (Fig. 3B). Incubation of MC3T3-E1 cells in CM from *Padi2 KO* cell lines #3–4 and #5–6 for 4 days did not induce an increase in *Cdkn1a* expression, but the cultivation for 8 days significantly increased *Cdkn1a* expression (Supplementary Figure S6A). These results strongly suggest that the decrease of PADI2 promotes the secretion of components of SASP, leading to propagation of cellular senescence gradually to surrounding cells. To determine which SASP factors are involved, we first investigated the expression changes of 45 SASP factors listed by Coppé J group by H_2_O_2_ treatment in RNA-seq data [12]. Among them, four SASP genes of *Ccl2, Ccl5, Ccl7* and *MMP-9* were significantly increased by H_2_O_2_ treatment (Supplementary Table S4). We validated the mRNA level of these upregulated genes via RT-qPCR (Fig. 3C). The major components of SASP, *Il1*α, *Il1*β, *Il6* and *Il18*, and the transcription factor Trp53 regulating SASP genes were also validated although they did not show a significant increase by H_2_O_2_ treatment in RNA-seq data (Fig. 3C). *Ccl2*, *5*, and *7* were significantly increased by the H_2_O_2_ treatment in both RNA-seq data and RT-qPCR, while very few transcripts of *Il1*α were detected in MC3T3-E1 cells and its expression was not induced by the H_2_O_2_ treatment. Unlike RNA-seq data, *MMP9* was not detected by RT-qPCR. Also, *Il1*β and *Il6* rather significantly decreased due to the H_2_O_2_ treatment in RT-qPCR, and *Trp53* was almost unchanged by H_2_O_2_ treatment compared with non-treated control in both RNA-seq data and RT-qPCR. *Il18* expression was slightly increased by H_2_O_2_ treatment in RT-qPCR. Furthermore, these three *Ccl2*, *5*, and *7* were significantly increased by the H_2_O_2_ treatment while satisfying our criteria among the CCL family in RNA-seq data (Supplementary file 9). Previous reports demonstrated roles of CCL2, 5, and 7 in senescence and aging. Circulating CCL2 levels increased in an age-dependent manner in wild-type mice, and that age-dependent increase was accelerated in Ercc1^−/Δ^ and Bubr1^H/H^ mouse models of progeria. Genetic and pharmacologic interventions that slow aging of Ercc1^−/Δ^ and WT mice lowered serum CCL2 levels significantly [26]. CCL5 increased in aged rat renal tissues compared with that in young and middle-aged groups [27] and CCL5 produced by senescent theca‐interstitial cells impairs follicle development and maturation during ovarian aging by promoting granulosa cell apoptosis [28]. The CCL7-CCL2-CCR2 axis was also suggested as a major cause of dysfunction of adipose tissue-derived stem cells from aged mice [29]. These previous reports and our data strongly indicate that CCL2, 5, and 7 might play a role in excessive ROS-induced senescence of osteoblasts rather than a typical SASP such as Il1α, Il1β, and Il6. Furthermore, *Padi2* knockdown also significantly increased the expression of *Ccl2, 5*, and *7* compared with that in siCont-transfected MC3T3-E1 cells (Fig. 3D). As was consistent with this finding, the secreted levels of CCL2, 5, and 7 were significantly elevated in the soup of *Padi2*-knockdown MC3T3-E1 cells compared with those in siCont-transfected cells (Fig. 3E). These mRNA expression and secreted protein levels of CCL2, 5, and 7 were significantly increased in both *Padi2* KO cell lines #3–4 and #5–6 compared with wild type (Supplementary Fig. S6B and C). Furthermore, the blockade of CCL2, 5, or 7 with each neutralizing antibody significantly inhibited *Padi2* knockdown-induced senescence of MC3T3-E1 cells, indicating that CCL2, 5, and 7 pay an important role in *Padi2* knockdown-induced osteoblast senescence (Fig. 3F). We also demonstrated that the knockdown of each of *Ccl2*, *5*, and *7* using specific siRNA against each gene effectively alleviated the H_2_O_2_- and *Padi2* knockdown-induced inhibition of osteogenic differentiation (Fig. 3G, H). Taken together, these findings suggest that osteoblast senescence caused by ROS-induced PADI2 reduction is promoted by the production of proinflammatory SASP, especially CCL2, CCL5, and CCL7 and the blockade of these SASP factors can alleviate the senescence and dysfunction of osteoblasts caused by excessive ROS and PADI2 depletion.

### The activation of NFκB RelA mediates ROS and *Padi2* knockdown-induced increase of CCL2, 5, and 7 SASP factors

**Fig. 4:**
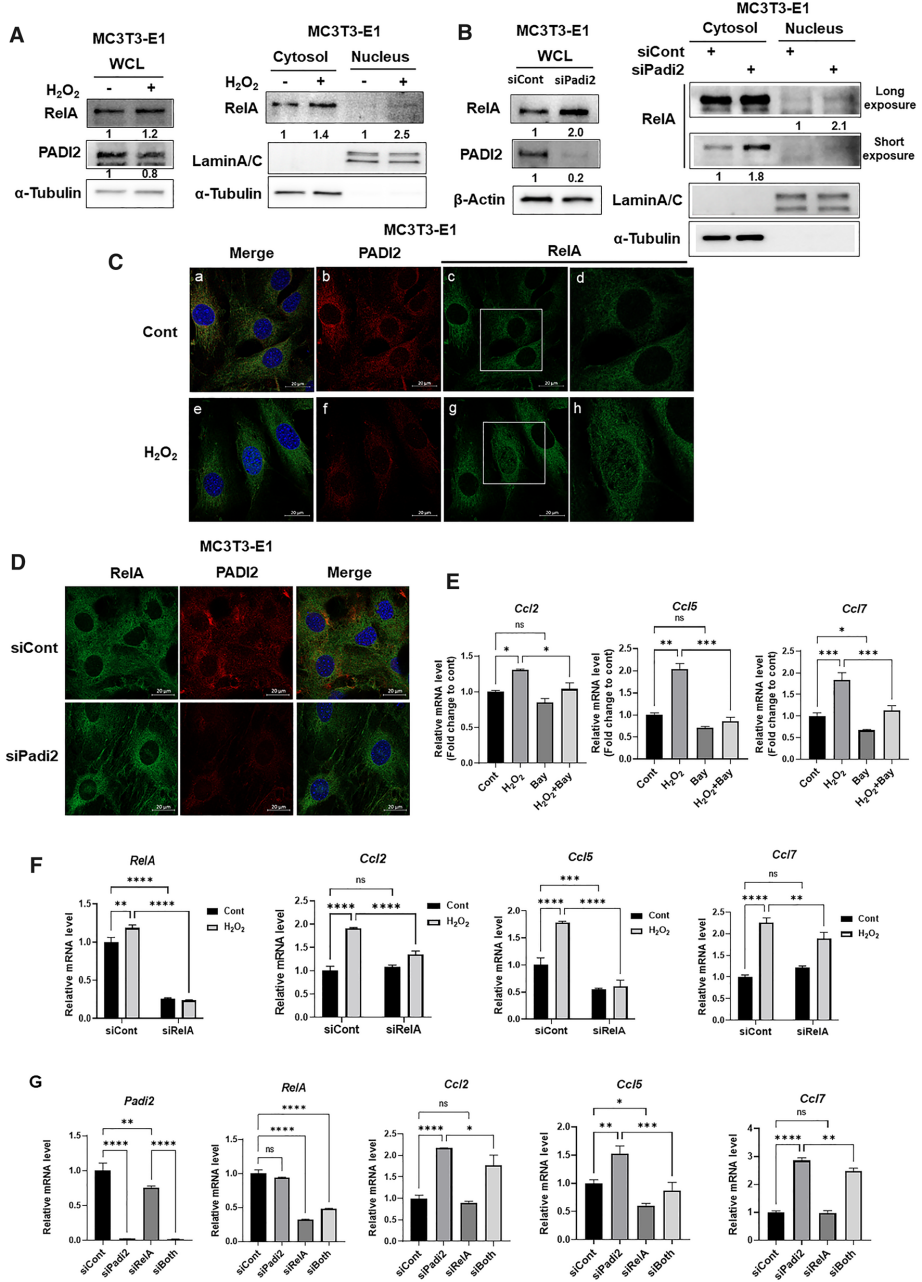
Activation of NFkB signaling mediates ROS- or *Padi2* knockdown-induced upregulation of the SASP. **A** and **B** When cells were fully confluent, MC3T3-E1 cells were treated with 100 μM H_2_O_2_ for 24 h (**A**) or cultured for additional 3 days after knockdown with siCont or siPadi2 (**B**), followed by the lysis of whole cell lysates and parallel subcellular fractionation. Western blot analysis was performed with the indicated antibodies. α-Tubulin or β-actin was used as an internal loading control. α-Tubulin and Lamin A/C were used as cytosolic and nuclear markers, respectively. Protein level is quantified using ImageJ and normalized with a-Tubulin, or β-actin, or Lamin A/C. After normalization, fold changes of treated/non-treated control are presented. The exposure time of membranes immunoblotted with anti-RelA was varied to distinguish band intensities (Long and short exposure). Three independent experiments were performed. **C** MC3T3-E1 cells were treated with 100 μM H_2_O_2_ for 24 h. Immunofluorescence staining was performed using the indicated antibodies and the nuclei were stained with DAPI. Boxed areas in c and g are enlarged and presented in the right column (d and h). Scale bar, 20 μm. Three independent experiments were performed. **D** Immunofluorescence staining of RelA and PADI2 in MC3T3-E1 cells knocked down with Padi2. Scale bar, 20 μm. Three independent experiments were performed. **E** When cells were fully confluent, MC3T3-E1 cells were treated with 100 μM H_2_O_2_ in combination with or without 2.5 μM Bay11-7082 in osteogenic medium for 4 days. The expression level of each gene was measured by RT-qPCR and the fold change in mRNA levels was calculated by normalization to *Gapdh*. Data are presented as the mean of three replicates; bars, ± SD; statistical significance was determined using one-way ANOVA for multiple comparisons, *P < 0.05, **P < 0.01, ***P < 0.001; ns, not significant. Three independent experiments were performed. **F** When MC3T3-E1 cells knocked down with siCont or siRelA were fully confluent, cells treated with 100 μM H_2_O_2_ in osteogenic medium for 4 days. RT-qPCR was performed. Data are presented as the mean of three replicates; bars, ± SD; statistical significance was determined using two-way ANOVA for multiple comparisons, **P < 0.01, ***P < 0.001, ***P < 0.001; ns, not significant. Three independent experiments were performed. **G** MC3T3-E1 cells were cultured in osteogenic medium for 4 days after knockdown of *Padi2* and/or *RelA*. All data of RT-qPCR are presented as the mean of three replicates; bars, ± SD; statistical significance was determined using one-way ANOVA for multiple comparisons, *P < 0.05, **P < 0.01, ***P < 0.001, ***P < 0.0001; ns, not significant. Three independent experiments were performed

The master transcription factor NFκB RelA/p65 (hereafter referred to as RelA) activates the transcription of several SASP genes [13]. TRRUST v2, a web-based prediction tool to search the transcriptional regulatory circuitry, also showed several CCL families including CCL2 and 5 as target genes of RelA [30]. When a NFκB signaling pathway is activated, RelA translocates to the nucleus to drive transcriptional regulation. It was also reported that H_2_O_2_ increases the DNA binding activity of NFκB and induces the translocation and phosphorylation of RelA [31]. We demonstrated that the treatment of H_2_O_2_ brought a slight increase in the protein level of RelA alongside the decrease in the PADI2 level (Fig. 4A, left panel) and furthermore, RelA, but not all, partially translocated from the cytoplasm to the nucleus in MC3T3-E1 cells by H_2_O_2_ treatment (Fig. 4A, right panel). Consistently, *Padi2* knockdown showed the two-fold increase in RelA protein level and induced the translocation of RelA to the nucleus, compared with the levels in the control (Fig. 4B), suggesting that PADI2 negatively regulates RelA protein level and its activity in osteoblasts. Immunofluorescence showed that the majority of PADI2 was detected predominantly in the cytoplasm of MC3T3-E1 osteoblasts and also confirmed that H_2_O_2_ treatment or *Padi2* knockdown induced the translocation of RelA from the cytoplasm to the nucleus, although the translocation of RelA into nucleus occurred only in a subset of cells not in all cells even under the same condition (Fig. 4C, D); this phenomenon seems to be in line with the stochastic theory of aging hypothesizing episodic events that happen through one’s life that cause random cell damage and accumulate over time, thus causing aging [32]. These results strongly indicate that the downregulation of PADI2 by H_2_O_2_ treatment activates the NFκB signaling pathway. Next, we investigated whether the increases in *Ccl2*, *5*, and *7* mRNA levels by the H_2_O_2_ treatment or *Padi2* knockdown can be abrogated by the blockade of the NFκB signaling pathway. Pharmacological inhibition of the NFκB signaling pathway using Bay11-7082, a potent inhibitor of IKKα, significantly reduced the H_2_O_2_-induced increases in *Ccl2*, *5*, and *7* mRNA levels in MC3T3-E1 cells (Fig. 4E). Furthermore, the knockdown of RelA significantly abolished the increases of *Ccl2*, *5*, and *7* mRNA levels by the H_2_O_2_ treatment or *Padi2* knockdown (Fig. 4F, G). Overall, our results showed that the H_2_O_2_ treatment or *Padi2* depletion upregulated *Ccl2*, *Ccl5*, and *Ccl7* SASP factors via the activation of NFκB RelA in osteoblasts.

### The inhibition of NFκB signaling alleviates the accelerated senescence and the reduced osteoblast differentiation caused by ROS or *Padi2* depletion

**Fig. 5:**
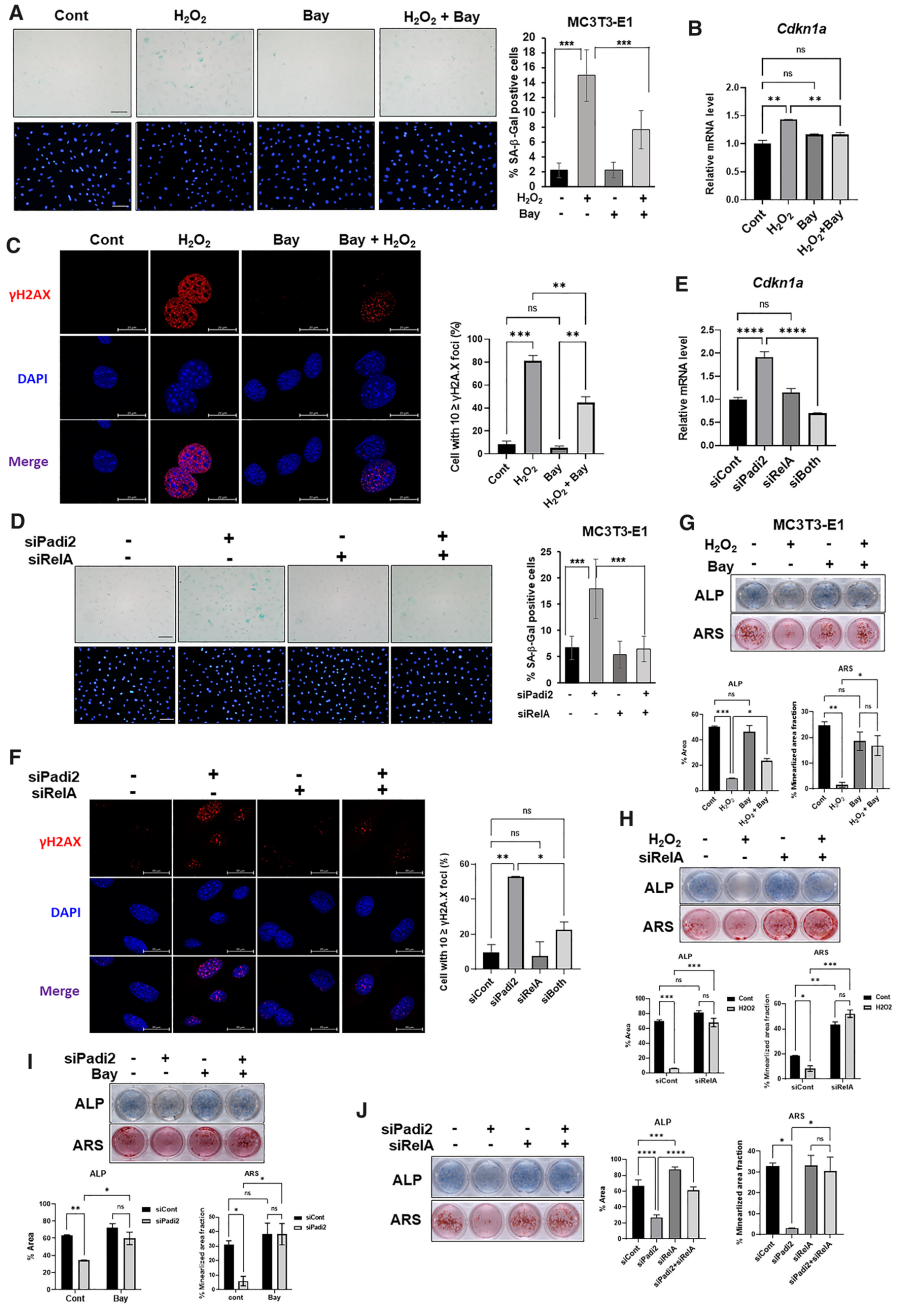
Inhibition of NFκB activity ameliorates ROS- or *Padi2* knockdown-accelerated cellular senescence and functional decline of osteoblasts. **A** Representative images of SA-β-Gal staining. MC3T3-E1 cells were treated with 100 μM H_2_O_2_ in combination with or without 2.5 μM Bay11-7082 for 24 h. Quantity of SA-β-Gal-positive cells was calculated as the percentage of SA-β-Gal-positive cells relative to the total cells stained with DAPI in the same field (10 fields/group). Magnification ×20, Scale bars 100 μm. Bars, mean ± SD; statistical significance was determined using one-way ANOVA for multiple comparisons, ***P < 0.001. Three independent experiments were performed. **B** When cells were fully confluent, MC3T3-E1 cells were treated with 100 μM H_2_O_2_ in combination with or without 2.5 μM Bay11-7082 in osteogenic medium for 4 days. *Cdkn1a* mRNA level was analyzed by RT-qPCR. Data are presented as the mean of three replicates; bars, ± SD; statistical significance was determined using one-way ANOVA for multiple comparisons, **P < 0.01; ns, not significant. Three independent experiments were performed. **C** Representative confocal microcopy images of γH2AX immunostaining. When cells were approximately 70–80% confluent, MC3T3-E1 cells were treated with 100 μM H_2_O_2_ in combination with or without 2.5 μM Bay11-7082 for 24 h, followed by γH2AX immunostaining (Red). The nuclei were stained with DAPI (Blue). Magnification × 100, scale bar, 20 μm. For quantification, the number of cells with ≥ 10 γH2AX foci was presented as a percentage. Bars, mean ± SD of two independent experiments; statistical significance was determined using one-way ANOVA for multiple comparisons, **P < 0.01, ***P < 0.001; ns, not significant. **D**, **E**, **F** MC3T3-E1 cells knocked down with siCont or siPadi2 in combination with siRelA were cultured for 3 days. **D** Representative images of SA-β-Gal staining. Quantity of SA-β-Gal-positive cells was calculated as the percentage of SA-β-Gal-positive cells relative to the total cells stained with DAPI in the same field (10 fields/group). Magnification ×20, Scale bars 100 μm. Bars, mean ± SD; statistical significance was determined using one-way ANOVA for multiple comparisons, ***P < 0.001. Three independent experiments were performed. **E** *Cdkn1a* qRT-PCR were performed. Data are presented as the mean of three replicates; bars, ± SD; statistical significance was determined using one-way ANOVA for multiple comparisons, ****P < 0.0001; ns, not significant. Three independent experiments were performed. **F** Representative confocal microcopy images of γH2AX immunostaining. Magnification × 100, scale bar, 20 μm. For quantification, the number of cells with ≥ 10 γH2AX foci was presented as a percentage. Bars, mean ± SD of two independent experiments; statistical significance was determined using one-way ANOVA for multiple comparisons, *P < 0.05, **P < 0.01; n.s., not significant. **G** MC3T3-E1 cells were treated with 100 μM H_2_O_2_ in combination with or without 2.5 μM Bay11-7082 in osteogenic medium and ALP and ARS staining was performed at day 4 and day 18, respectively. **H** and **I** MC3T3-E1 cells knocked down with siRelA or siPadi2 were treated with 100 μM H_2_O_2_ or 2.5 μM Bay11-7082 in osteogenic medium and ALP and ARS staining was performed at day 4 and day 18, respectively. **J** MC3T3-E1 cells knocked down with siPadi2 in combination with or without siRelA were incubated in osteogenic medium and ALP and ARS staining was performed at day 4 and day 18, respectively. Quantification of each staining was performed by ImageJ. Bars, mean ± SD; statistical significance was determined using one-way ANOVA for multiple comparisons, *P < 0.05, **P < 0.01,***P < 0.001, ****P < 0.0001; ns, not significant. Three independent experiments were performed

To determine whether the blockade of NFκB signaling can relieve the ROS-accelerated cellular senescence of osteoblasts, MC3T3-E1 cells were treated with H_2_O_2_ in combination with or without Bay11-7082. SA-β-Gal staining showed that the H_2_O_2_-induced cellular senescence was significantly decreased by simultaneous treatment with H_2_O_2_ and Bay11-7082 (Fig. 5A). Consistent with this, *Cdkn1a* mRNA level increased by H_2_O_2_ treatment was significantly alleviated by blocking NFκB signaling using Bay11-7082 (Fig. 5B), and also the increase of cells with the accumulation of γH2AX foci by H_2_O_2_ treatment was significantly relieved by co-treatment with Bay11-7082 (Fig. 5C). Similarly, it was shown that the number of SA-β-Gal-positive cells increased by *Padi2* knockdown was significantly reduced by Bay11-7082 treatment (Supplementary Figure S7A). The increase of *Cdkn1a* mRNA level and cells with the accumulation of γH2AX foci by *Padi2* knockdown was correlatively alleviated by Bay11-7082 treatment (Supplementary Figure S7B and C). SA-β-Gal staining showed that *RelA* knockdown also relieved the senescence of osteoblasts induced by *Padi2* knockdown (Fig. 5D). Consistent with this finding, *Cdkn1a* mRNA level and cells with the accumulation of γH2AX foci increased by *Padi2* knockdown was significantly reduced by blocking NFκB signaling using *RelA* knockdown (Fig. 5E, F). Next, we examined whether the dysfunction of osteoblasts by ROS or PADI2 reduction could also be ameliorated by blocking NFκB signaling. As shown in Fig. 5G and H, the H_2_O_2_-induced inhibition of osteogenic differentiation of MC3T3-E1 cells was relieved by simultaneous treatment with Bay11-7082 or *RelA* siRNA. The decline of osteoblast differentiation by *Padi2* knockdown was also significantly restored by the co-treatment of Bay 11–7087 or *RelA* knockdown (Fig. 5I, J). Taken together, these results strongly indicate that the inhibition of NFκB signaling effectively alleviates the ROS- or PADI2-depletion-induced senescence and dysfunction of osteoblasts.

## Discussion

The bone undergoes a continuous remodeling process in which an old bone is resorbed and replaced with newly formed bone. With aging, however, this remodeling balance becomes overthrown, resulting in a decreased bone mass. The decline in bone mass is associated with reduced bone strength, resulting in osteoporosis [33]. Although bone mineral density (BMD) is an important predictor of subsequent fracture risk, age itself is also a major determinant factor of the fracture risk, independent of BMD [33, 34]. Age-related bone loss accompanies change in both trabecular and cortical bone microstructures. A major and the most important age-related bone loss occurs in trabecular bone caused by the thinning of the trabeculae and by disruption of the trabecular microstructure and loss of trabecular elements. The histomorphometric analysis of bone biopsies from elderly men and women demonstrated that the impairment of bone formation by osteoblasts is the major cause of age-related bone loss compared to bone resorption by osteoclasts [2]. Increased osteoblast senescence contributes to age-related functional decline in osteoblasts together with increased apoptosis [35] and impaired osteoblast differentiation [36]. Oxidative stress by excessive ROS is one of the main causes of cellular senescence and can adversely affect bone homeostasis, including the suppression of osteoblast activity and enhancement of osteoclast activity that lead to osteopenia [37–40]. While studies have mainly focused on revealing the mechanisms of action of ROS to activate osteoclasts, the mechanisms by which ROS regulate osteogenic differentiation and the function of osteoblasts have been poorly studied. Although cortical bone loss in which osteocytes reside also contributes to age-related bone loss, this study focused on oxidative stress-induced senescence of osteoblasts responsible for trabecular bone formation, which account for the most important part of age-related bone loss. For the first time, the present study found that the reduction of PADI2 by excessive ROS induced the senescence and dysfunction of osteoblasts. As for its regulatory mechanism, our study also revealed that the decrease of PADI2 increased the expression and secretion of SASP via NFκB activation, leading to the propagation of cellular senescence and dysfunction of osteoblasts.

The PADI enzymes have been implicated in a variety of physiological processes, including epigenetic and transcriptional regulation and tissue differentiation [7]. In this study, PADI2 was identified and verified as a key factor regulating the ROS-induced senescence and dysfunction of osteoblasts. Of the five PADI isoforms, PADI2 and PADI4 are the most studied, and the excessive citrullination of target proteins by their abnormal enzyme activity is one of the main causes of some diseases, such as rheumatoid arthritis (RA) and many cancer types. Together with PADI4 [41], the protein level of PADI2 was reported to be highly increased in RA synovial tissues compared with that in samples from patients with osteoarthritis and ankylosing spondylitis, and some SNPs of the PADI2 gene were shown to be significantly associated with the presence of RA [42]. PADI2 expression is reported to be increased in many types of tumor tissues including breast, cervical, esophageal, colon, lung, gastric, liver, and etc. [43]. Another studies also show that PADI2 expression is positively associated with the progression of endometrial carcinoma and ovarian cancer and the downregulating PADI2 suppressed the proliferation of these cancer cell lines [44, 45]. On the basis of the pathophysiology by PADI-mediated hypercitrullination, the inhibition of these enzymes has been suggested as a therapeutic strategy for RA and cancers. In addition, PADI2 plays an important role in cell differentiation and function in normal physiological conditions. Falcao’s team showed that the *Padi2* expression increased upon oligodendrocyte (OL) differentiation and that the reduction of PADI2 activity hindered OL differentiation [46]. Our study also showed that PADI2 expression increased gradually during osteoblast differentiation and its reduction by siRNA resulted in the inhibition of osteoblast differentiation. On the basis of our study and that of other groups, although the hyperactivation of PADI2 is mostly associated with disease pathology, PADI2 may be essential for the differentiation and function of specific cell types such as osteoblasts and OL in normal physiology. However, the roles and regulatory mechanisms of PADI enzymes in cellular senescence and aging as well as in osteogenic differentiation have not yet been reported. To the best of our knowledge, the present study is the first to demonstrate that the ROS-induced reduction of PADI2 or *Padi2* knockdown not only induces cellular senescence, but also leads to a marked functional decline of osteoblasts. Our results imply that PADI2 is an essential factor for maintaining cell homeostasis from oxidative stress and for maintaining osteoblast differentiation and function. PADI2-mediated citrullination may also have important roles in osteoblasts. Therefore, further studies that reveal the citrullinome in osteoblast differentiation and senescence to understand the functional regulation of osteoblasts, and that provide new therapeutic targets for the treatment of age-associated bone diseases and the enhancement of bone regeneration in the elderly are required.

Recent report shows that PADI2 overexpression suppresses the proliferation of HCT116 colon cancer cell line and PADI2 is downregulated in colon cancer compared with matching normal tissue [47]. This contradicts with the role of PADI2 shown in previously reported other cancer types and our results that both *Padi2* knockdown and CRISPR/Cas9-mediated *Padi2* knockout significantly inhibit the proliferation of osteoblasts (Fig. 2B and Supplementary Figure S5B). PADI2 expression patterns in colon cancer also appear to differ between the two study groups [43, 47]. The opposing role of PADI2 in cell proliferation may be due to the cell-type-specific roles of PADI2, but this has not been clearly elucidated. If the same target plays the opposite role in different tissues, the targeted therapy may cause unexpected side effects, so in-depth studies are needed.

The mechanisms that drive senescence in post-mitotic cells and the contribution of post-mitotic cell senescence to tissue degeneration during aging are emerging areas of interest [48]. Evidence for post-mitotic senescence can be also found in the bone and may contribute to age-related bone disease [49, 50]. Osteocytes are long-living post-mitotic cells embedded in cortical bone and they have dendritic processes connecting them to other osteocytes and surface osteoblasts. Conversely, osteoblasts are short-lived bone forming post-mitotic cells, continuously replenished by osteogenic stem cells [51]. In our study, to mimic chronic ROS accumulation during aging, fully confluent osteoblasts were applied chronically with non-lethal concentration of H_2_O_2_ during osteoblast differentiation. Chronic treatment with H_2_O_2_ did not induce apoptosis, but significantly inhibited the osteogenic differentiation of MC3T3-E1 cells accompanying cellular senescence (Supplementary Fig. S1), indicating that oxidative stress induces post-mitotic senescence of osteoblasts.

The senescence growth arrest is often triggered by a persistent DNA damage response (DDR) elicited by intrinsic and extrinsic stress such as ROS and ionizing radiation [23, 24]. *Padi2* knockdown induces the significant accumulation of γH2AX foci in osteoblasts and increased the percentage of cells with γH2AX foci (Fig. 2C). The accumulation of γH2AX foci also drastically increased in CRISPR/Cas9-edited *Padi2* KO cell line and the percentage of damaged cells was over 80% (Supplementary Figure S5C). These data strongly suggest that PADI2 play an important role in maintaining genomic integrity and protecting the genome from oxidative damage. Further studies are needed to pinpoint the mechanisms by which persistent DNA damage due to the reduction of PADI2 induces post-mitotic senescence of osteoblasts and PADI2 contributes to genomic stability.

In GO analysis in Fig. 1B, GO term (GO:0030500) of “regulation of bone mineralization” was simultaneously shown in both groups of increased gene set on Day1 and decreased gene set on Day4. Genes upregulated on day 1, *ECM1*, *Bglap*, *Bglap2*, *Bglap3*, and *PTK2B* are included and genes downregulated on day 4 include *Phospho1*, *Bglap*, *Bglap2*, *Bglap3*, and *Ifitm5* in this GO term. *ECM1* and *PTK2B* were reported as negative regulators for bone formation [52, 53]. On the contrary, both *Phospho1* and *Ifitm5* play positive roles in bone formation [54, 55]. These results suggest that ROS induces negative regulators of bone formation and simultaneously suppresses positive regulators during osteoblast differentiation. However, three genes, *Bglap*, *Bglap2*, and *Bglap3,* show up commonly in both groups. When we validated this phenomenon with RT-qPCR, the qPCR results were consistent with the RNA seq results (Data not shown). Osteocalcin (OCN) is encoded by a single gene (*BGLAP*) in human, while mice have a cluster of three genes (*Bglap*, *Bglap2*, and *Bglap3*) within a 25 kb genomic region [56, 57]. Because the role of OCN in bone has been still controversial, this phenomenon shown in our data is hard to be clearly explained at this time.

The SASP can act as a negative effector responsible for chronic inflammation and age-linked diseases in old organisms [58, 59]. Our results show that CCL2, 5, and 7, but not a typical SASP such as IL1α, IL1β and IL6, play important roles in osteoblast senescence induced by stresses such as ROS and PADI2 reduction (Fig. 3). CCL2 has been reported to have a role in the activation of bone resorption. The PTH-induced osteoblastic expression of CCL2 facilitates the recruitment, differentiation, and fusion of osteoclast precursors [60]. Elevated Ca^2+^ in the bone remodeling process stimulates CCL5 secretion from osteoblasts [61]. In turn, CCL5 secreted from osteoclasts or osteoblasts promotes the chemotaxis of both cells and also prevents the apoptosis of osteoblasts [61]. CCL7 can also induce osteoclast migration, resorption activity, adhesion, and survival [62]. On the basis of these studies, CCL2, CCL5, and CCL7 secreted from senescent osteoblasts due to oxidative stress or PADI2 reduction may increase the differentiation, recruitment, and adhesion of osteoclasts while increasing the survival of senescent osteoblasts, thereby further promoting bone resorption and the deterioration of bone function with aging. Although the involvement of other members of the secretome of the SASP cannot be ruled out, our results strongly indicate that CCL2, CCL5, and CCL7 act as key SASP factors in the oxidative stress- or *Padi2*-loss-induced cellular senescence of osteoblasts. In addition, these results suggest that the inflammation induced by an increase in these SASP factors due to oxidative stress may be a major cause of age-related bone loss.

Here, we observed that ROS or *Padi2* loss significantly increased the NFκB RelA protein level and also activated NFκB signaling through the translocation of RelA into the nucleus (Fig. 4A–D), suggesting that PADI2 directly or indirectly regulates RelA protein level. However, in Fig. 4G, when *Padi2* is knocked down, there is no change in mRNA expression of *RelA*. On the contrary, when *RelA* is knocked down, there is a reduction in *Padi2* mRNA, suggesting that RelA regulates *Padi2* gene expression. This confliction of regulatory relationship between PADI2 and RelA might be explained in this way: PADI2 can regulate RelA protein on the post-translational level but not on transcription level while RelA can be involved in regulation of *Padi2* transcription directly or indirectly as a transcription factor. In accordance with our data, PADI2 appears to negatively regulate RelA through citrullination in the normal physiological state of osteoblasts, which induces its conformational change, promoting proteasomal degradation. Therefore, the reduction of PADI2 in senescent osteoblasts may decrease the citrullination of RelA, resulting in the prevention of its degradation. Similarly, it has been reported that PADI2 suppresses NFκB activity via interaction with IKKγ, an upstream activator of RelA, and its citrullination in the macrophage cell line RAW264.7 [63]. The study at hand also suggested that PADI2 acts as a negative regulator of the NFκB signaling pathway. However, a recent study reported that PADI4 directly citrullinates NFκB RelA in RA, which improves the interaction between RelA and importin α3, thereby increasing the translocation of RelA into the nucleus and its transcriptional activity [64]. Although the findings of that previous study conflict with our data on the regulation of RelA by PADI2, this suggests the possibility that PADI2 can also directly regulate RelA through citrullination at the post-translational stage in osteoblasts. Furthermore, these conflicting roles of PADIs in NFκB regulation may be due to differences in cell type among osteoblasts, macrophages, and neutrophils, or in the unique role of each PADI enzyme. Therefore, further studies are needed on how PADI2 regulates NFκB RelA in osteoblasts in a normal or senescent state.

**Fig. 6:**
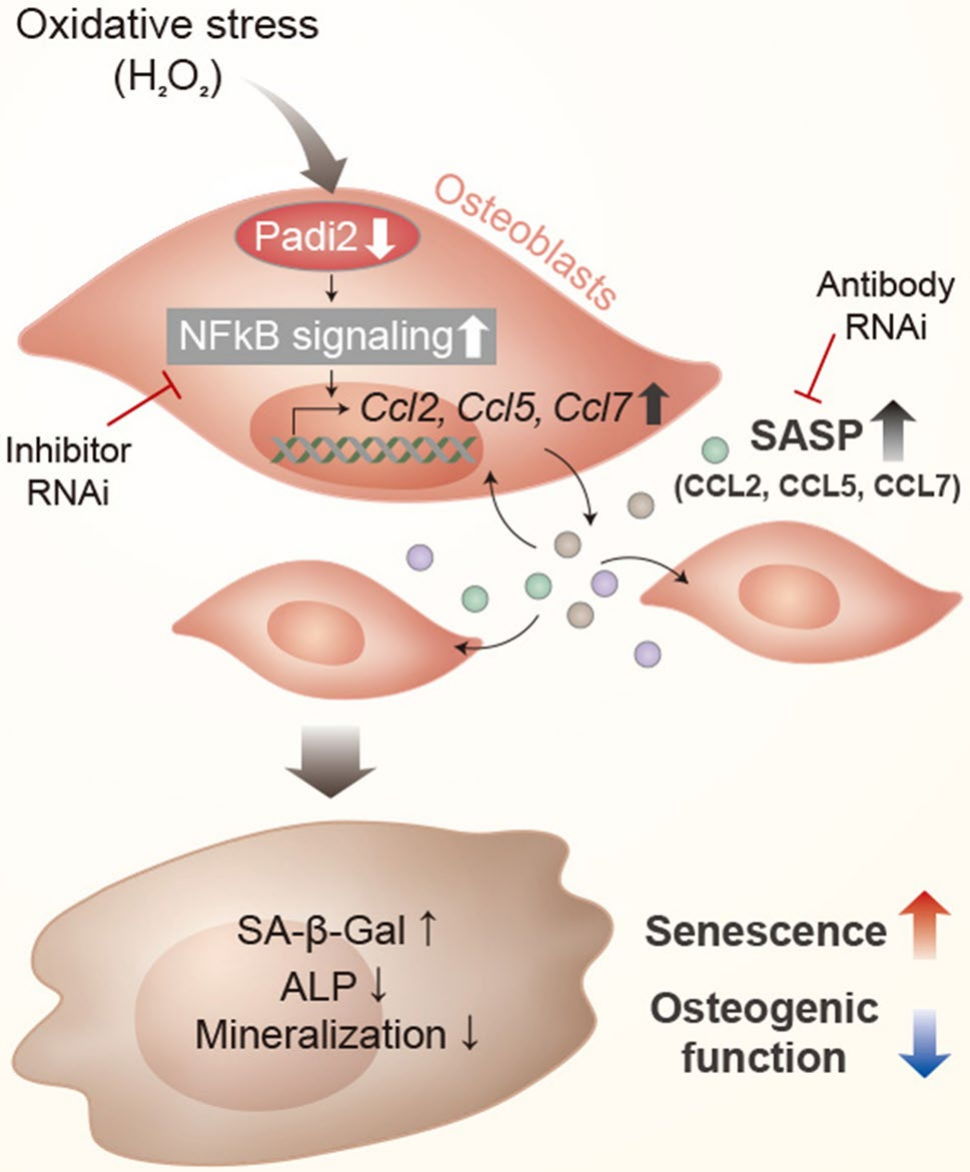
Schematic diagram depicted in the mechanism of oxidative stress-accelerated senescence and dysfunction of osteoblasts. The reduction of PADI2 by oxidative stress induces upregulation of   CCL2, 5, and 7 known as the SASP, through the activation of NFκB signaling, leading to making a senescent environment and propagating cellular senescence to surrounding cells. Targeting these regulatory processes may be an effective way to prevent or treat excessive ROS-promoted cellular senescence and aging-related bone diseases

In conclusion, this study demonstrates for the first time the role of PADI2 as a protector against ROS-accelerated cellular senescence of osteoblasts. The mechanism of oxidative stress-induced senescence and dysfunction of osteoblasts was schematically summarized in Fig. 6. The reduction of PADI2 by oxidative stress leads to upregulation of the SASP through the activation of NFκB signaling, creating a senescent environment and propagating cellular senescence to surrounding cells. Targeting these regulatory processes may be an effective way to prevent or treat excessive ROS-promoted cellular senescence and aging-related bone diseases.

### Supplementary Information

Below is the link to the electronic supplementary material.

Supplementary file1 (DOCX 2076 KB)

Supplementary file2 (XLSX 40544 KB)

Supplementary file3 (XLSX 15 KB)

Supplementary file4 (XLSX 9 KB)

Supplementary file5 (XLSX 19 KB)

Supplementary file6 (XLSX 15 KB)

Supplementary file7 (XLSX 17 KB)

Supplementary file8 (XLSX 16 KB)

Supplementary file9 (XLSX 21 KB)

## Data Availability

The data that support the findings of this study are available from the corresponding author upon reasonable request. As such, the authors will follow guidance provided by the journal for sharing the data.

## Abbreviations

ROS: Reactive oxygen species
PADI2: Peptidyl arginine deiminase 2
SASP: Senescence-associated secretory phenotype
NFκB: Nuclear factor kappa B
CCL2: C–C motif chemokine ligand 2
CCL5: C–C motif chemokine ligand 5
CCL7: C–C motif chemokine ligand 7
IL-1α: Interleukin-1α
IL-1β: Interleukin-1β
GSEA: Gene set enrichment analysis
