# Supplementary material for: ROS-induced PADI2 downregulation accelerates cellular senescence via the stimulation of SASP production and NFκB activation
Source: Cell Mol Life Sci. 2022 Feb 26;79(3):155. doi: 10.1007/s00018-022-04186-5 (PMC8882118; doi:10.1007/s00018-022-04186-5)
Supplement: Supplementary file 1 — Supplementary file1 (DOCX 2076 KB) [file 18_2022_4186_MOESM1_ESM.docx]

**Supplementary Data**

**ROS-induced PADI2 downregulation accelerates cellular senescence via the stimulation of SASP production and NFκB activation**

Hyun-Jung Kim^1^, Woo-Jin Kim^1^, Hye-Rim Shin^1^, Hee-In Yoon^1^, Jae-I Moon^1^, Eunji Lee^1^, Jin-Muk Lim^2,3^, Young-Dan Cho^4^, Mi-Hye Lee^5^, Hong-Gee Kim^2^, Hyun-Mo Ryoo^1,^*

^1^Department of Molecular Genetics and Dental Pharmacology, School of Dentistry and Dental Research Institute, Seoul National University, Seoul, South Korea

^2^Biomedical Knowledge Engineering Laboratory, School of Dentistry and Dental Research Institute, Seoul National University, Seoul, South Korea

^3^Alopax-Algo, Co. Ltd, Seoul, South Korea.

^4^Department of Periodontology, School of Dentistry and Dental Research Institute, Seoul National University, Seoul, South Korea

^5^Department of Biology and Center for Cell Reprogramming, Georgetown University, Washington DC, USA

**Supplementary experimental procedures**

***Padi2* knockout cell construction by CRISPR–Cas9 gene editing system**

*Padi2* gene knockout by CRISPR–Cas9 gene editing was performed using Padi2 Mouse Gene Knockout Kit (CRISPR) (Cat#KN512746; Origene), in accordance with the manufacturer’s protocol. MC3T3-E1 cells were transfected with Cas9-containing plasmid pCas-Guide, which included guide RNAs targeting the first exon of mouse Padi2 together with linear donor DNA containing LoxP-EF1A-tGFP-P2A-Puro-LoxP cassette to be used as a reporter for positive transfection. Single-cell fluorescence-activated cell sorting (FACS) was carried out for the selection of individual transfected cells, which were then grown into colonies under puromycin selection (5 μg/mL). Western blotting was subsequently used to confirm protein level depletion in selected *Padi2* KO clones including #3-4 and #5-6. For wild-type (wt) control, MC3T3-E1 cells were transfected with pCMV6-A-puro vector (Cat#PS100025; Origene) and then colonies were selected under puromycin. Selected clones were pooled together to rule out clonal artifacts and used as wt control cells.

**BrdU proliferation assay**

Cell proliferation was quantified in a 96-well plate (2×10^4^ cells/well) using a colorimetric BrdU Cell Proliferation ELISA Kit (ab126556; Abcam), in accordance with the manufacturer’s instructions. Results are presented as BrdU incorporation fold change (absorbance at 450 nm relative to wt control as indicated) and all experiments were repeated at least three times and are presented as mean ± SD.

**Gene Set Enrichment Analysis (GSEA)**

For Gene Ontology analysis, GSEA (Broad Institute) were performed. We followed the standard procedure as described by GSEA user guide and used curated gene set C5 of the Molecular Signature Database version 7.4 (http://www.broadinstitute.org/gsea/index.jsp).

**Statistical analysis**

To ensure data reliability, all experiments were performed as at least two or three independent experiments with three replicates. For statistical analyses, *P* values were calculated by *t-*test (when comparing only two groups) or one-way ANOVA or two-way ANOVA (when comparing more than two groups) in GraphPad Prism 9 with default parameters. All results are expressed as the mean ± SD, and differences were considered significant at *P* < 0.05. *P* values are as follows: **P* < 0.05; ***P* < 0.01; ****P* < 0.001; *****P* < 0.0001.


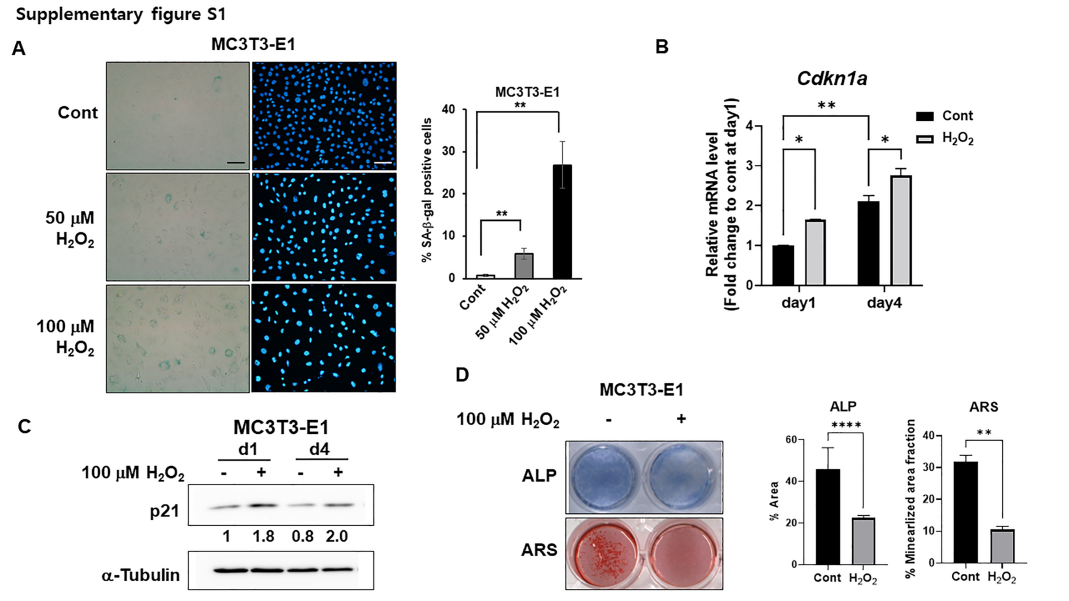


**Supplementary Figure S1. ROS induce cellular senescence and the decline of osteogenic differentiation in MC3T3-E1 cells** (A) Representative images of SA-β-Gal staining. MC3T3-E1 cells were treated with the indicated concentration of H_2_O_2_ for 24-48 hours and then SA-β-Gal staining was performed. SA-β-Gal-positive cells in each group were calculated as the percentage of SA-β-Gal-positive cells relative to the total cells stained with DAPI in the same field (10 fields/group). Magnification ×20, Scale bars 100 μm. Bars, mean±SD; statistical significance was determined using one-way ANOVA for multiple comparisons, ****P* < 0.001. Three independent experiments were performed. (B) When cells were fully confluent, MC3T3-E1 cells were treated with or without 100 μM H_2_O_2_ in osteogenic medium for each indicated day. *Cdkn1a* mRNA level was determined by RT-qPCR and the fold change in mRNA levels was calculated by normalization to *Gapdh*. Data are presented as the mean of three replicates; bars, ±SD; statistical significance was determined using two-way ANOVA for multiple comparisons, **P* < 0.05, ***P* < 0.01. Three independent experiments were performed. (C) Western blot analysis of p21 expression levels at the indicated time points during osteogenic differentiation of MC3T3-E1. α-Tubulin was used as an internal loading control. Protein level is quantified using ImageJ and normalized with a-Tubulin. After normalization, fold changes of treated /non-treated control are presented. (D) When cells were fully confluent, MC3T3-E1 cells were treated with or without 100 μM H_2_O_2_ in osteogenic medium and ALP and ARS staining was performed at day 4 and day 18, respectively. Quantification of each staining was performed by ImageJ. Bars, ±SD; statistical significance was determined using two-tailed Student t-test, ***P* < 0.01, *****P* < 0.0001. Three independent experiments were performed.


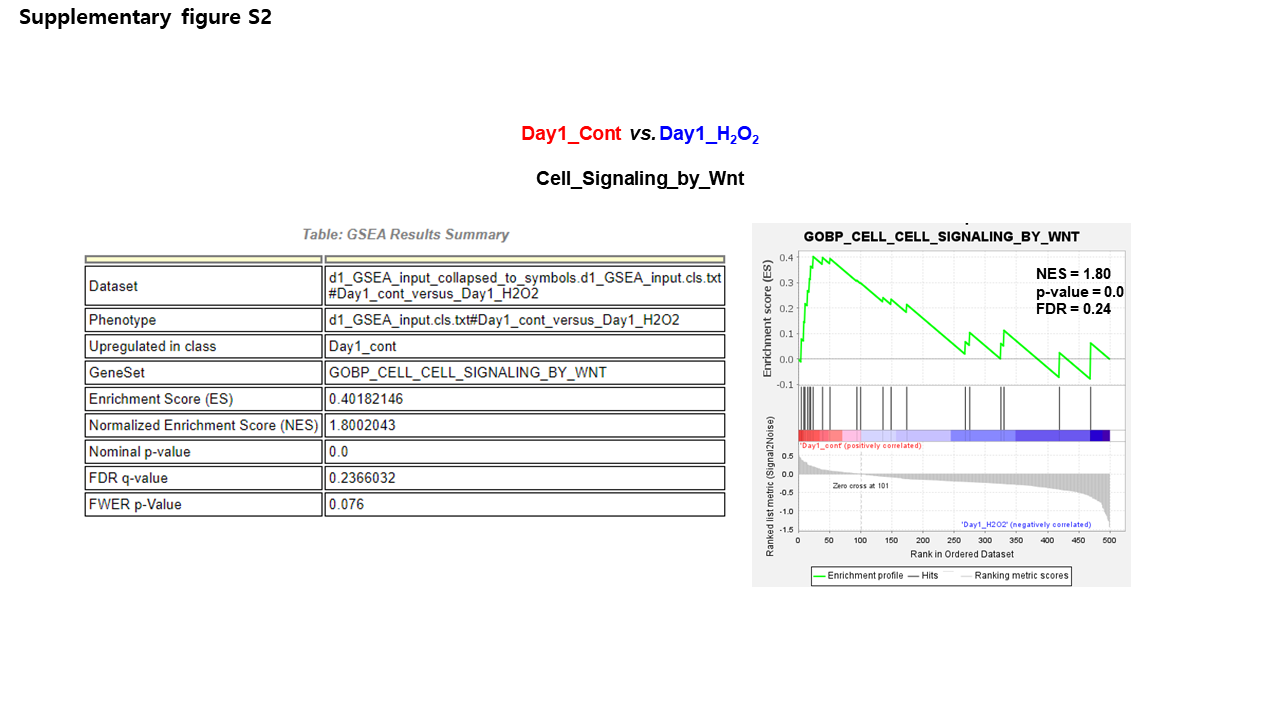


**Supplementary Figure S2. GSEA analysis of Day1 cont versus Day1 H_2_O_2_-treated group.** GSEA reveals negative enrichment of Day1 H_2_O_2_-treated group in the gene set of cell signaling by Wnt. Normalized enrichment score (NES), nominal p-value, and FDR are shown.


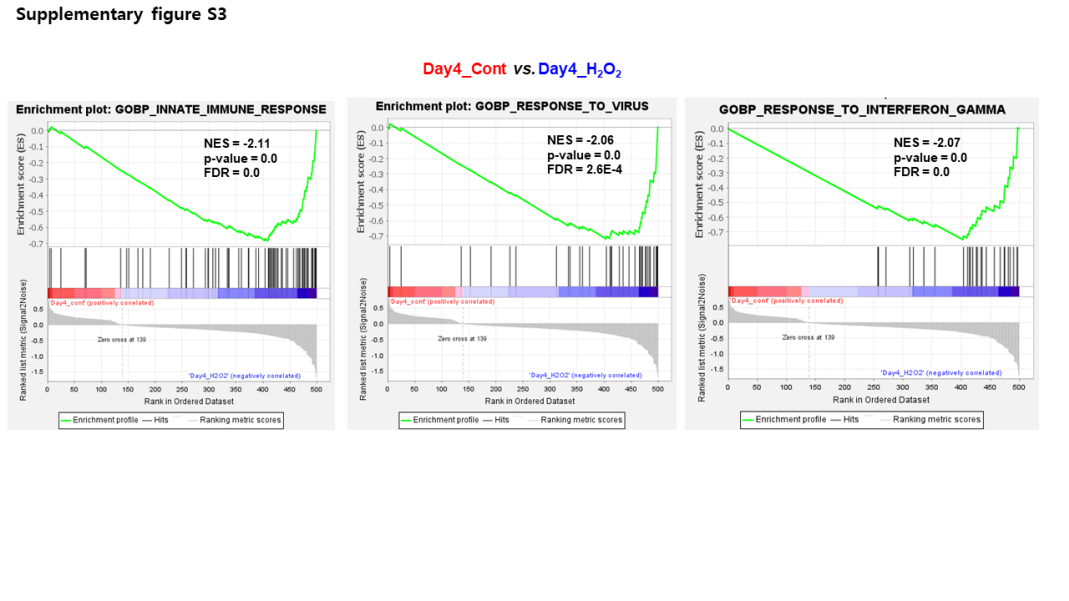


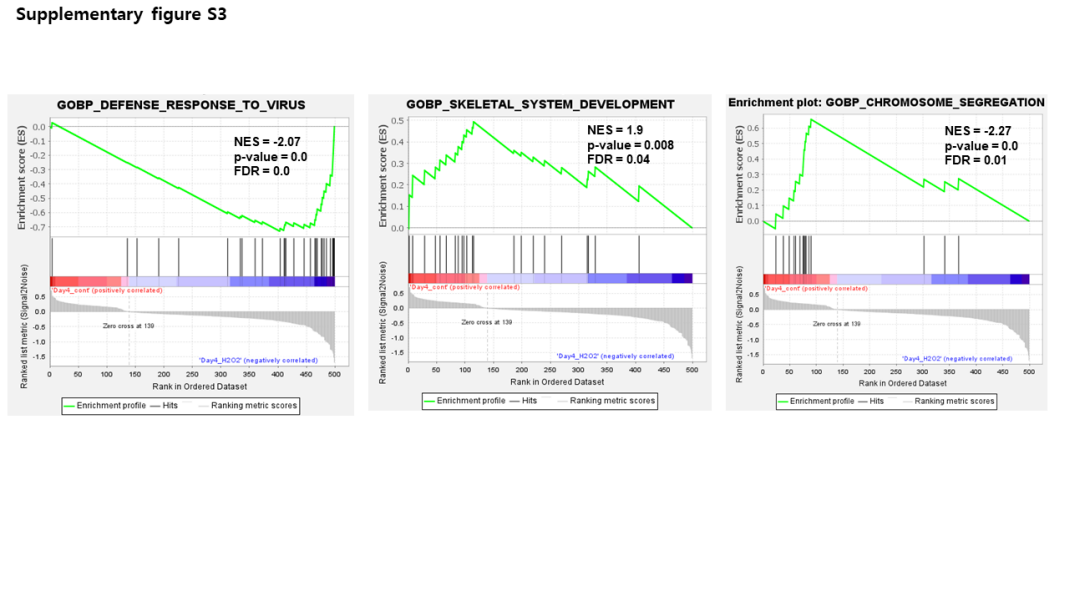


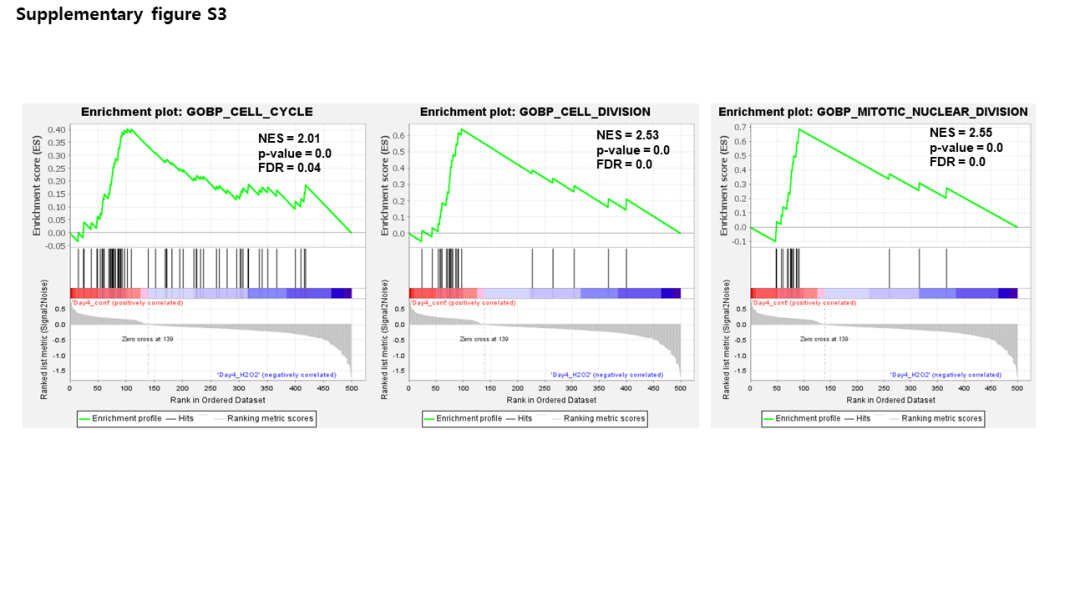


**Supplementary Figure S3. GSEA analysis of Day4 cont versus Day1 H_2_O_2_-treated group.** GSEA using the C5 reference gene set reveals positive enrichment of Day4 H_2_O_2_-treated group in the gene sets of innate immune response, response to virus, response to interferon gamma, and defense response to virus. Negative enrichment of the gene sets of skeletal system development, chromosome segregation, cell cycle, cell division, and mitotic nuclear division are shown in Day4 H_2_O_2_-treated group. Normalized enrichment score (NES), nominal p-value, and FDR are shown.


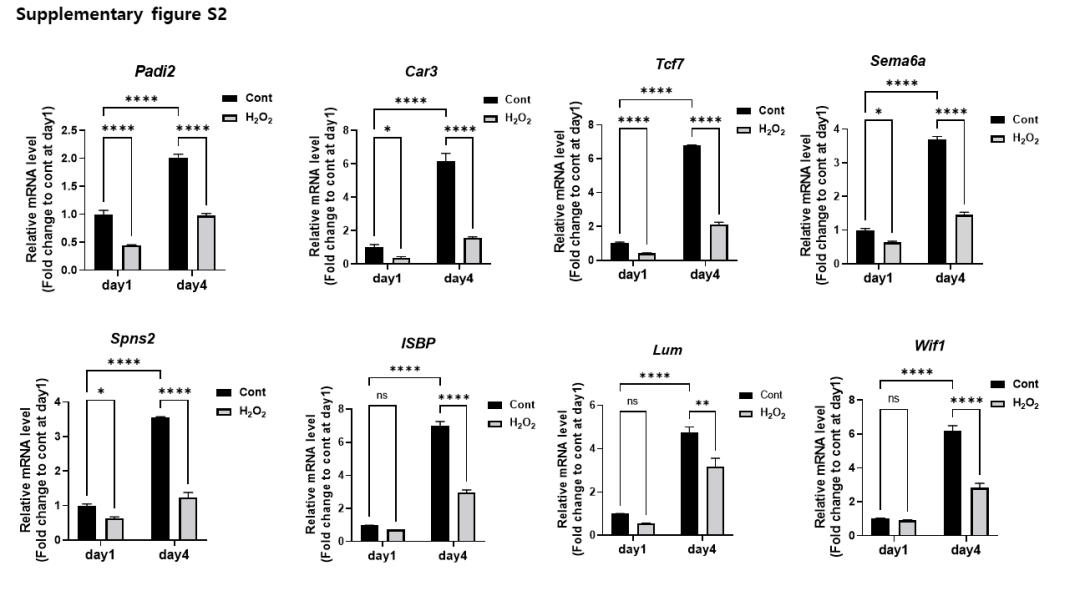


**Supplementary Figure S4. Validation of mRNA expression of 8 commonly downregulated coding genes at day1 and day4 in RNA-seq by RT-qPCR.** When cells were fully confluent, MC3T3-E1 cells were treated with or without 100 μM H_2_O_2_ in osteogenic medium for each indicated day. mRNA level was determined by RT-qPCR with each specific primer set and the fold change in mRNA levels was calculated by normalization to *Gapdh*. Data are presented as the mean of three replicates; bars, ±SD; statistical significance was determined using two-way ANOVA for multiple comparisons, **P* < 0.05, ***P* < 0.01. Three independent experiments were performed.


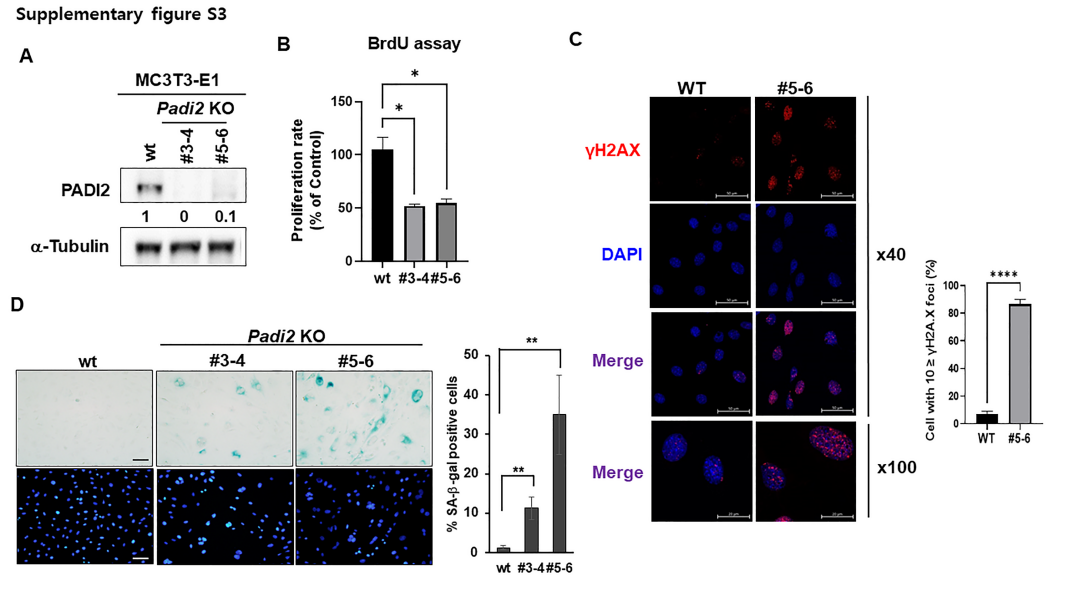


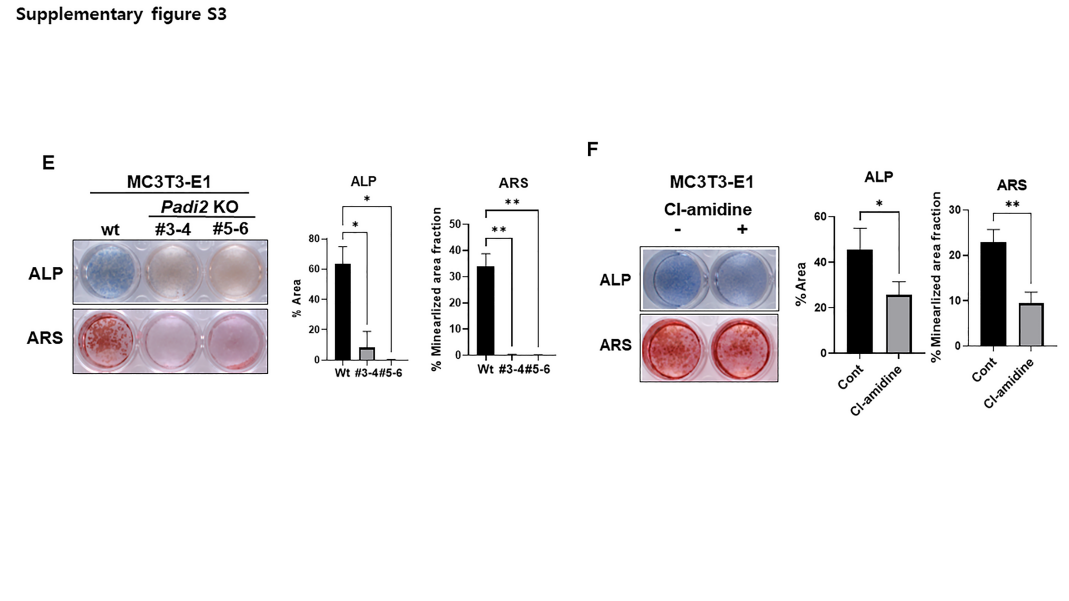
**Supplementary Figure S5. CRISPR–Cas9-mediated *Padi2* knockout confirms the role of PADI2 in cellular senescence and osteogenic differentiation of MC3T3-E1 cells** (A) Validation of *Padi2* knockout (KO) in CRISPR–Cas9-mediated *Padi2* KO cell clones (#3-4 and #5-6) by western blot analysis. Protein level is quantified using ImageJ and normalized with a-Tubulin. After normalization, fold changes to wt control are presented. (B) BrdU incorporation assay showed that *Padi2* KO cell clones exhibited lower proliferation rates than wild-type MC3T3-E1 cells (wt). Data are presented as the mean of three replicates; bars, ±SD; statistical significance was determined using one-way ANOVA for multiple comparisons, **P* < 0.05. Three independent experiments were performed. (C) Representative confocal microcopy images of γH2AX immunostaining. Cells plating on coverslips were cultured for 2 days (approximately 60-70% confluency), followed by γH2AX staining (Red). The nuclei were stained with DAPI (Blue). Scale bar, 50μm for magnification x40, 20 μm for magnification x100. In the graph on the right, the number of cells with ≥10 rH2AX foci was presented as a percentage. Bars, mean±SD of two independent experiments; statistical significance was determined by two-tailed Student t-test, *****P* < 0.0001. (D) Representative images of SA-β-Gal staining of wt and *Padi2* KO cell clones. SA-β-Gal-positive cells in each group were calculated as the percentage of SA-β-Gal-positive cells relative to the total cells stained with DAPI in the same field (8-10 fields/group). Magnification ×20, Scale bars 100 μm. Bars, mean±SD; statistical significance was determined using one-way ANOVA for multiple comparisons, ***P* < 0.01. Three independent experiments were performed. (E) *Padi2* KO clones and wt cells were cultured in osteogenic medium and ALP and ARS staining was performed at day 4 and day 18, respectively. Quantification of each staining was performed by ImageJ. Bars, mean±SD; statistical significance was determined using one-way ANOVA for multiple comparisons, **P* < 0.05, ***P* < 0.01. Three independent experiments were performed. (F) When cells were fully confluent, MC3T3-E1 cells were treated with or without 5 μM Cl-amidine in osteogenic medium and ALP and ARS staining was performed at day 4 and day 18, respectively. Quantification of each staining was performed by ImageJ. Bars, mean±SD; statistical significance was determined using two-tailed Student t-test, **P* < 0.05, ***P* < 0.01. Three independent experiments were performed.


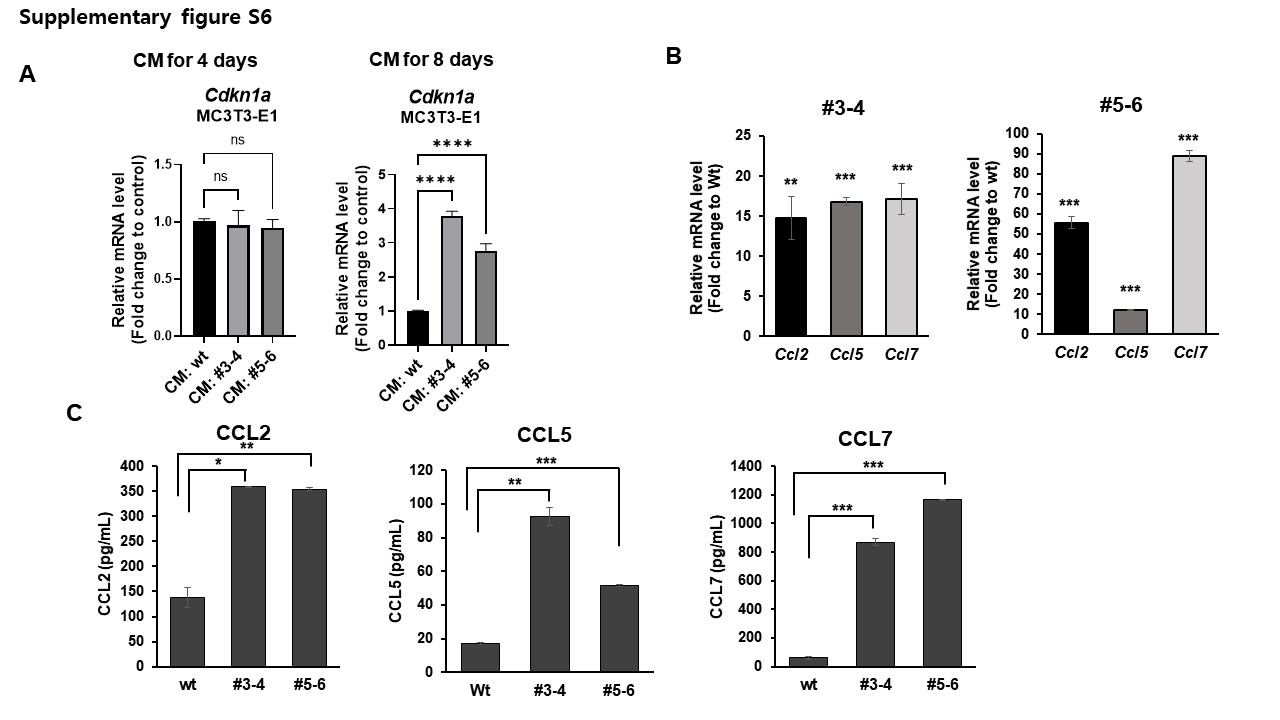
**Supplementary Figure S6. mRNA and secretion levels of CCL2, CCL5, and CCL7 are dramatically increased in CRISPR–Cas9-mediated *Padi2* KO cell clones compared with those in wild-type MC3T3-E1 cells** (A) MC3T3-E1 cells were cultivated under Padi2 KD-CM or siCont-CM for 4 and 8 days, followed by performing RT-qPCR of *Cdkn1a.* Data for RT-qPCR are presented as the mean of three replicates; bars, ±SD. statistical significance was determined using one-way ANOVA for multiple comparisons, *****P* < 0.0001; n.s., not significant. Two independent experiments were performed. (B) *Ccl2*, *Ccl5*, and *Ccl7* mRNA levels in wt and *Padi2* KO cell clones were analyzed by RT-qPCR. The data normalized to *Gapdh* are presented as fold changes of *Padi2* KO clones relative to wt. Data are presented as the mean of three replicates; bars, ±SD; *P* values for comparisons between each *Padi2* KO cell clone and wt by two-tailed Student’s t-test, ***P* < 0.01, ****P* < 0.001. (C) Conditioned media from wt and two *Padi2* KO cell clones at day 4 were collected and the levels of CCL2, CCL5, and CCL7 were measured by ELISA. Data are presented as the mean of three replicates; bars, ±SD; statistical significance was determined using one-way ANOVA, **P* < 0.05, ***P* < 0.01, ****P* < 0.001. Three independent experiments were performed.


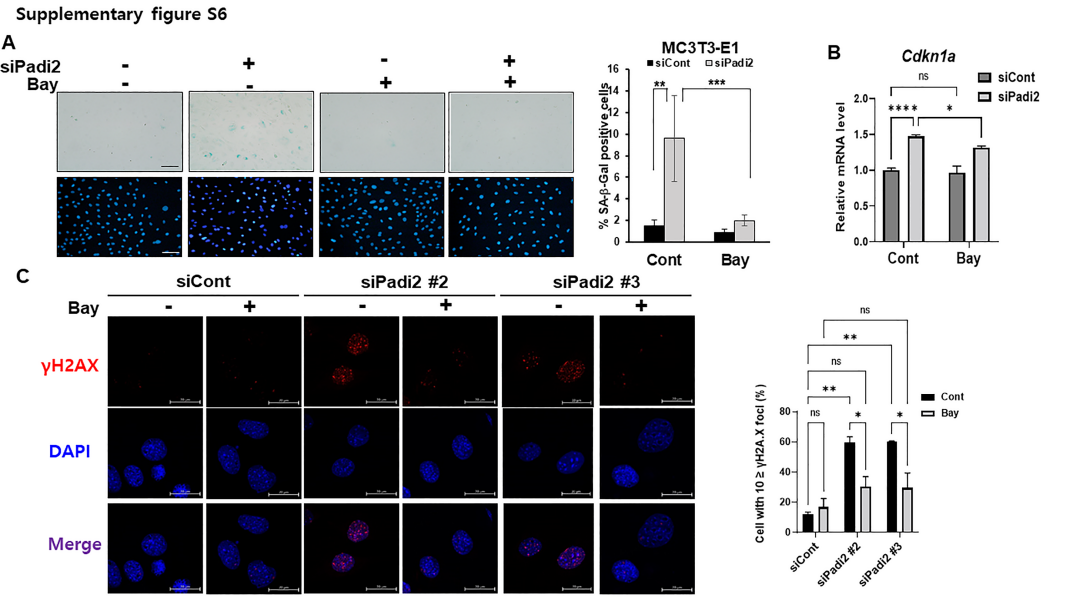


**Supplementary Figure S7. Inhibition of NFκB activity by Bay 11-7082 ameliorates *Padi2* knockdown-accelerated senescence of osteoblasts.** (A) Representative images of SA-β-Gal staining. MC3T3-E1 cells knocked down with siCont or siPadi2 were treated with 2.5 μM Bay 11-7082 for 24 h. Quantity of SA-β-Gal-positive cells was calculated as the percentage of SA-β-Gal-positive cells relative to the total cells stained with DAPI in the same field (8-10 fields/group). Magnification ×20, Scale bars 100 μm. Bars, mean±SD; statistical significance was determined using two-way ANOVA for multiple comparisons, ***P* < 0.01, ****P* < 0.001. Three independent experiments were performed. (B) When MC3T3-E1 cells knocked down with siCont or siPadi2 were fully confluent, cells were treated with 100 μM H_2_O_2_ in combination with or without 2.5 μM Bay 11-7082 in osteogenic medium for 4 days. *Cdkn1a* mRNA level was analyzed by RT-qPCR. Data are presented as the mean of three replicates; bars, ±SD; statistical significance was determined using two-way ANOVA for multiple comparisons, **P* < 0.05, *****P* < 0.0001; ns, not significant. Three independent experiments were performed. (C) Representative confocal microcopy images of γH2AX immunostaining. MC3T3-E1 cells knocked down with siCont or siPadi2 were treated with 2.5 μM Bay 11-7082 for 24 h., followed by γH2AX immunostaining (Red). The nuclei were stained with DAPI (Blue). Magnification x100, scale bar, 20 μm. For quantification, the number of cells with ≥10 rH2AX foci was presented as a percentage. Bars, mean±SD of two independent experiments; statistical significance was determined using two-way ANOVA for multiple comparisons, **P* < 0.05, ***P* < 0.01; ns, not significant.

**Supplementary Table S1. Commonly upregulated genes on Days 1 and 4 (fold change, 1.5; log2-normalized read counts of ≥5; *P* <0.05)**

|  |  | **Fold change**  **(H_2_O_2_/Cont)** | | ***P-*value** | |
| --- | --- | --- | --- | --- | --- |
|  | **Gene symbol** | **Day 1** | **Day 4** | **Day 1** | **Day 4** |
| 1 | **Ces2e** | 8.245 | 5.716 | 0.000 | 0.003 |
| 2 | **Slit1** | 3.513 | 2.497 | 0.034 | 0.001 |
| 3 | **Siglecg** | 3.430 | 2.275 | 0.008 | 0.046 |
| 4 | **Slc29a4** | 3.366 | 4.663 | 0.026 | 0.011 |
| 5 | **Vnn1** | 3.296 | 3.159 | 0.021 | 0.005 |
| 6 | **Plcd4** | 3.286 | 2.414 | 0.029 | 0.027 |
| 7 | **Iqcg** | 2.725 | 2.102 | 0.003 | 0.020 |
| 8 | **Gm37090** | 2.653 | 1.790 | 0.021 | 0.048 |
| 9 | **1700007K13Rik** | 2.595 | 2.446 | 0.015 | 0.041 |
| 10 | **Procr** | 2.471 | 2.180 | 0.035 | 0.029 |
| 11 | **Trim5** | 2.387 | 1.948 | 0.008 | 0.020 |
| 12 | **Zbp1** | 2.326 | 10.445 | 0.047 | 0.003 |
| 13 | **Svop** | 2.273 | 1.680 | 0.001 | 0.007 |
| 14 | **Oasl2** | 2.172 | 11.977 | 0.024 | 0.014 |
| 15 | **Gm13415** | 2.140 | 2.598 | 0.012 | 0.022 |
| 16 | **Nrg1** | 2.034 | 2.284 | 0.003 | 0.014 |
| 17 | **Sptbn5** | 1.954 | 1.652 | 0.043 | 0.043 |
| 18 | **Ntn4** | 1.913 | 1.779 | 0.044 | 0.011 |
| 19 | **Cyp4f14** | 1.901 | 1.688 | 0.039 | 0.016 |
| 20 | **Irf7** | 1.897 | 6.344 | 0.038 | 0.001 |
| 21 | **Sp110** | 1.891 | 2.059 | 0.012 | 0.047 |
| 22 | **Ckmt1** | 1.885 | 1.746 | 0.006 | 0.001 |
| 23 | **H2-T23** | 1.870 | 1.507 | 0.017 | 0.005 |
| 24 | **Mmp9** | 1.840 | 1.966 | 0.041 | 0.004 |
| 25 | **Pappa** | 1.760 | 1.767 | 0.041 | 0.022 |
| 26 | **Podn** | 1.730 | 1.550 | 0.009 | 0.019 |
| 27 | **Oas2** | 1.692 | 13.021 | 0.018 | 0.049 |
| 28 | **Htra3** | 1.688 | 1.731 | 0.006 | 0.004 |
| 29 | **Parp14** | 1.675 | 2.643 | 0.010 | 0.038 |
| 30 | **Trim34a** | 1.648 | 2.285 | 0.037 | 0.003 |
| 31 | **Ly6a** | 1.614 | 1.666 | 0.012 | 0.006 |
| 32 | **Pdk4** | 1.606 | 1.547 | 0.010 | 0.006 |
| 33 | **Ifi203** | 1.574 | 3.321 | 0.013 | 0.022 |
| 34 | **Kitl** | 1.569 | 1.811 | 0.015 | 0.026 |
| 35 | **Gbp2** | 1.552 | 2.565 | 0.032 | 0.018 |
| 36 | **Trim12a** | 1.537 | 1.504 | 0.025 | 0.025 |
| 37 | **Nacad** | 1.536 | 1.516 | 0.046 | 0.028 |
| 38 | **Ptk2b** | 1.526 | 1.534 | 0.001 | 0.006 |
| 39 | **Plscr2** | 1.521 | 1.623 | 0.017 | 0.008 |
| 40 | **Id4** | 1.514 | 2.089 | 0.049 | 0.017 |

**Supplementary Table S2. Commonly downregulated genes on Days 1 and 4 (fold change, 1.5; log2-normalized read counts of ≥5; *P* <0.05)**

|  |  | **Fold change**  **(H_2_O_2_/Cont)** | | ***P-*value** | |
| --- | --- | --- | --- | --- | --- |
|  | **Gene symbol** | **Day 1** | **Day 4** | **Day 1** | **Day 4** |
| 1 | **Padi2** | 0.457 | 0.564 | 0.006 | 0.028 |
| 2 | **Lum** | 0.485 | 0.638 | 0.015 | 0.038 |
| 3 | **Car3** | 0.492 | 0.341 | 0.049 | 0.008 |
| 4 | **Spns2** | 0.523 | 0.405 | 0.041 | 0.010 |
| 5 | **Tcf7** | 0.580 | 0.403 | 0.029 | 0.004 |
| 6 | **Wif1** | 0.630 | 0.533 | 0.008 | 0.011 |
| 7 | **Ibsp** | 0.642 | 0.463 | 0.045 | 0.013 |
| 8 | **Sema6a** | 0.657 | 0.474 | 0.023 | 0.001 |
| 9 | **H19** | 0.389 | 0.325 | 0.006 | 0.022 |
| 10 | **Gm8343** | 0.584 | 0.650 | 0.006 | 0.033 |

**Supplementary Table S3. Genes regulated in opposite directions on Days 1 and 4 (fold change, 1.5; log2-normalized read counts of ≥5; *P* <0.05)**

|  |  | **Fold change**  **(H_2_O_2_/Cont)** | | ***P-*value** | |
| --- | --- | --- | --- | --- | --- |
|  | **Gene symbol** | **Day 1** | **Day 4** | **Day 1** | **Day 4** |
| 1 | **Bglap** | 2.222 | 0.537 | 0.021 | 0.005 |
| 2 | **Bglap3** | 2.063 | 0.557 | 0.043 | 0.026 |
| 3 | **Bglap2** | 2.617 | 0.562 | 0.042 | 0.007 |

**Supplementary Table S4. Changes in the senescence-associated secretory phenotype (SASP) factors by Day4 H_2_O_2_ treatment compared with control in MC3T3-E1 cells.**

| **SASP factors** | | **Secretory profile of senescent cells**  **(**Coppé JP et al., 2010) | **Our RNA-seq data**  **Day 4 H_2_O_2_ treatment** |
| --- | --- | --- | --- |
| **Interleukins (IL)** | IL-1α | up | n.s.**^a^** |
|  | IL-1β | up | no reads**^b^** |
|  | IL-6 | up | n.s. |
|  | IL-7 | up | n.s |
|  | IL-13 | up | no reads |
|  | IL-15 | up | n.s. |
| **Chemokines**  **(CXCL, CCL)** | Cxcl1 | up | no reads |
|  | Cxcl2 | up | no reads |
|  | Cxcl3 | up | no reads |
|  | Cxcl8 (IL-8) | up | not found**^c^** |
|  | Cxcl15 | up | no reads |
|  | **Ccl2 (MCP-1)** | up | **up** |
|  | **Ccl5 (RANTES)** | up | **up** |
|  | **Ccl7** | up | **up** |
|  | Ccl8 (MCP-2) | up | no reads |
|  | Ccl11(Eotaxin) | up | no reads |
|  | Ccl16 (HCC-4) | up | not found |
|  | Ccl20 (MIP-3a) | up | no reads |
|  | Ccl26 (Eotaxin-3) | up | no reads |
| **Growth factors and regulators** | Amphiregulin (Areg) | up | n.s. |
|  | Epiregulin (Ereg) | up | n.s. |
|  | EGF | up or no change | n.s |
|  | bFGF | up | n.s |
|  | HGF | up | n.s. |
|  | KGF(FGF7) | up | n.s |
|  | VEGF | up | n.s |
|  | Angiogenin (Ang) | up | not found |
|  | SCF | up | not found |
|  | SDF-1 | up or no change | not found |
|  | PIGF | up | n.s |
|  | IGFBP-2 | up | n.s |
|  | IGFBP-3 | up | no reads |
|  | IGFBP-4 | up | n.s |
|  | IGFBP-6 | up | n.s. |
|  | IGFBP-7 | up | n.s |
| **Proteases and regulators** | MMP-1 | up | no reads |
|  | MMP-3 | up | no reads |
|  | **MMP-9** | up | **up** |
|  | MMP-10 | up | no reads |
|  | MMP-12 | up | no reads |
|  | MMP-13 | up | n.s |
|  | MMP-14 | up | n.s |
|  | TIMP-1 | down or no change | n.s |
|  | TIMP-2 | up | n.s |
|  | Cathepsin B (Ctsb) | up | n.s |

**^a^** n.s., not significant

**^b^** No reads indicate that raw read count is 0.

**^c^** Not found means when the gene is not listed in RNA-seq data.

**Supplementary Table S5. Primer sequences used for RT-qPCR analysis in this study (forward, FOR; reverse, REV)**

| **Gene** | **Sequences for primers** |
| --- | --- |
| ***Padi1*** | FOR: CGTGCAGAAATGCATCGACT |
|  | REV: ATGTCCACGATGTCGCTCTC |
| ***Padi2*** | FOR: GACAAGGTCACTGTCAACTACTATGAA |
|  | REV: TTGTTCTTCTCCACCTCTCCAT |
| ***Padi3*** | FOR: CCTAGGCCGGCATGTCTCTA |
|  | REV: CTCAGGAACCGCCCCATAAA |
| ***Padi4*** | FOR: GGCTACACAACCTTCGGCAT |
|  | REV: GCTGCTTTCACCTGTAGGGT |
| ***Padi6*** | FOR: GTGGCTAGCTTGGTAAGCCC |
|  | REV: TGCACACTTGCTGATGTCCAA |
| ***PADI1*** | FOR: TCCCTGAAGATGCCTACCCA |
|  | REV: CACCCTTGGGCACATCACTGT |
| ***PADI2*** | FOR: GCAGGCTGCTGGAGAAGG |
|  | REV: CGCTGTAGACATCGGTCCAG |
| ***PADI3*** | FOR: AGTCCAACACCAGCATGTCG |
|  | REV: CCTCAGGCACTGACCCATAAA |
| ***PADI4*** | FOR: GTTTAGGGTCAGACAGTCCTGG |
|  | REV: AGATGTGAGTAGTGGCACATGC |
| ***PADI6*** | FOR: CCTGACCTGTTGCGGATGAT |
|  | REV: AGCAGGTCCCCTTGATTTGG |
| ***Cdkn1a*** | FOR: AGATCCACAGCGATATCCAGAC |
|  | REV: ACCGAAGAGACAACGGCACACT |
| ***Il1α*** | FOR: CGAAGACTACAGTTCTGCCATT |
|  | REV: GACGTTTCAGAGGTTCTCAGAG |
| ***Il1β*** | FOR: GCAACTGTTCCTGAACTCAACT |
|  | REV: ATCTTTTGGGGTCCGTCAACT |
| ***Il6*** | FOR: CTTCCATCCAGTTGCCTTCTTG |
|  | REV: AATTAAGCCTCCGACTTGTGAAG |
| ***Il18*** | FOR: GTGAACCCCAGACCAGACTG |
|  | REV: CCTGGAACACGTTTCTGAAAGA |
| ***Ccl2*** | FOR: TGCTGACCCCAAGAAGGAAT |
|  | REV: GAAGTGCTTGAGGTGGTTGTG |
| ***Ccl5*** | FOR: AGATCTCTGCAGCTGCCCTCA |
|  | REV: GGAGCACTTGCTGCTGGTGTAG |
| ***Ccl7*** | FOR: CTTTCAGCATCCAAGTGTGGG |
|  | REV: ATGCTATAGCCTCCTCGACC |
| ***MMP9*** | FOR: TTGACAGCGACAAGAAGTGG |
|  | REV: GCCATTCACGTCGTCCTTAT |
| ***Trp53*** | FOR: GGGCGTAAACGCTTCGAGAT |
|  | REV: TCAGGTAGCTGGAGTGAGCC |
| ***RelA*** | FOR: GAGTCTCCATGCAGCTACGG |
|  | REV: TTCTCTTCAATCCGGTGGCG |
| ***Lum*** | FOR: CTCTTGCCTTGGCATTAGTCG  REV: GGGGGCAGTTACATTCTGGTG |
| ***Car3*** | FOR: TGACAGGTCTATGCTGAGGGG  REV: CAGCGTATTTTACTCCGTCCAC |
| ***Wif1*** | FOR: TCTGGAGCATCCTACCTTGC  REV: ATGAGCACTCTAGCCTGATGG |
| ***IBSP*** | FOR: ATGGAGACGGCGATAGTTCC  REV: CTAGCTGTTACACCCGAGAGT |
| ***Sema6a*** | FOR: ACAGCCTGCCCCCTAAAGT  REV: AGCTCCTCTTATATTCGAGCCC |
| ***Spns2*** | FOR: GCTGCTGCCATCCTGAGTTT  REV: CAGGTAGCCAAAGATGGGGG |
| ***Tcf7*** | FOR: AGCTTTCTCCACTCTACGAAC  REV: AATCCAGAGAGATCGGGGGTC |
| ***Gapdh*** | FOR: GGCCTCACCCCATTTGATGT |
|  | REV: CATGTTCCAGTATGACTCCACTC |
| ***GAPDH*** | FOR: TTCGACAGTCAGCCGCATCTTCTT |
|  | REV: GCCCAATACGACCAAATCCGTTGA |
